# Mating system and speciation I: Accumulation of genetic incompatibilities in allopatry

**DOI:** 10.1371/journal.pgen.1010353

**Published:** 2022-12-15

**Authors:** Lucas Marie-Orleach, Christian Brochmann, Sylvain Glémin

**Affiliations:** 1 Natural History Museum, University of Oslo, Oslo, Norway; 2 CNRS, Université Rennes 1, ECOBIO (écosystèmes, biodiversité, évolution) – UMR 6553, Rennes, France; 3 Department of Ecology and Genetics, Evolutionary Biology Centre, Uppsala University, Uppsala, Sweden; University of Western Ontario, CANADA

## Abstract

Self-fertilisation is widespread among hermaphroditic species across the tree of life. Selfing has many consequences on the genetic diversity and the evolutionary dynamics of populations, which may in turn affect macroevolutionary processes such as speciation. On the one hand, because selfing increases genetic drift and reduces migration rate among populations, it may be expected to promote speciation. On the other hand, because selfing reduces the efficacy of selection, it may be expected to hamper ecological speciation. To better understand under which conditions and in which direction selfing affects the build-up of reproductive isolation, an explicit population genetics model is required. Here, we focus on the interplay between genetic drift, selection and genetic linkage by studying speciation without gene flow. We test how fast populations with different rates of selfing accumulate mutations leading to genetic incompatibilities. When speciation requires populations to pass through a fitness valley caused by underdominant and compensatory mutations, selfing reduces the depth and/or breadth of the valley, and thus overall facilitates the fixation of incompatibilities. When speciation does not require populations to pass through a fitness valley, as for Bateson-Dobzhanzky-Muller incompatibilities (BDMi), the lower effective population size and higher genetic linkage in selfing populations both facilitate the fixation of incompatibilities. Interestingly, and contrary to intuitive expectations, local adaptation does not always accelerate the fixation of incompatibilities in outcrossing relative to selfing populations. Our work helps to clarify how incompatibilities accumulate in selfing *vs*. outcrossing lineages, and has repercussions on the pace of speciation as well as on the genetic architecture of reproductive isolation.

## Author summary

Hermaphroditic organisms may use their male gametes to fertilise their own female gametes, but species vary greatly in how much they self-fertilise. Self-fertilisation (selfing) induces many genetic modifications in populations, which may ultimately affect the rates at which lineages diversify. Here we aim to build predictions on how selfing affects the rate at which hybrid incompatibility arises between geographically isolated populations. We developed theoretical models in which populations with variable selfing rates may fixate mutations leading to hybrid incompatibility. First, we explored scenarios wherein hybrid incompatibility is caused by mutations whose fixations necessitates a population to experience temporally deleterious effects (*i.e*., a fitness valley), and found that selfing reduces the breadth and depth of the fitness valley and thereby overall facilitates the accumulation of such mutations. Second, we explored scenarios wherein hybrid incompatibility is caused by interactions between derived alleles of different genes (*i.e*., BDMi). We found that selfing reduces the manifestation of BDMi within population, and thereby facilitates their fixation. This effect prevails even in the face of local adaptation. Thus, our study clarifies how fast hybrid incompatibilities should accumulate in selfing populations, and helps to understand how new species are expected to arise in selfing lineages.

## Introduction

*Species belonging to the same section or species group usually cross freely in woody plants and perennial herbs, but are usually separated by incompatibility barriers in annual herbs. […] It is possible, therefore, that the correlation […] between sterility and life form is a reflection of a more fundamental relationship between type of breeding system and the formation of sterility barriers*” [1].

The wide variety of mating systems observed in animals, plants, fungi and algae has multiple ecological and evolutionary consequences that might impact higher-level evolutionary processes such as species extinction and speciation. For instance, hermaphroditic species vary in their rate of self-fertilisation—spanning from obligate outcrossing via all degrees of mixed mating to predominant selfing species [2–4]—and this has long been argued to affect macroevolutionary processes [5–9]. Because selfing tends to reduce both the genetic diversity and the adaptive potential of populations, selfing lineages have been argued to be ‘evolutionary dead-ends’ as they can be expected to go extinct at faster rates than outcrossing lineages [5, 8, 10, 11]. However, the study of the macroevolutionary effects of selfing has mostly focused on extinction, whereas the effects of selfing on speciation have received less attention, both empirically and conceptually (but see [7, 9]).

Given that fully selfing species are extremely rare, perhaps even non-existent [2, 3], we consider the biological species concept to be applicable to selfing species too. Thus, the process of speciation in both selfing and outcrossing lineages involves the gradual build-up of genetic incompatibilities between two diverging entities. The mechanisms and pace of reproductive isolation (RI) may however differ across mating systems.

Phylogenetic studies of plants that include taxa with different mating systems can be used to assess whether rates of species diversification, and potentially of speciation and extinction, differ between selfing and outcrossing lineages (*e.g*., [12–15]). In the Solanaceae, outcrossing is enforced by a self-incompatibility mechanism, but this has broken down several times, resulting in independently derived self-compatible lineages. Compared to the self-incompatible lineages (*i.e*., obligate outcrossers), the self-compatible ones (*i.e*., potential selfers) show lower net diversification rates [16] which, interestingly, are likely to be caused by higher rates of both speciation and species extinction in the selfing lineages [12]. Phylogenetic studies in the Primulaceae [13] and in the Onagraceae [15] suggest that young selfing taxa experience a burst of speciation that fades away with time (*i.e*., a ‘senescing diversification rate’ [17]). In contrast, evidence in the Polemoniaceae is mixed: alternative phylogenetic methods provide either positive or no associations between selfing and speciation rates [14].

The effects of selfing on speciation have also been tested using experimental crosses, to address whether RI between populations or species evolves at different rates in selfing *vs*. outcrossing species. To our knowledge however, there are only a few such studies. In the Arctic flora, intraspecific crosses between geographically isolated populations of eight predominantly selfing species resulted in F1 hybrids with low pollen fertility and seed set, whereas no reduced fertility was observed in the single outcrossing species for which successful crosses could be made [18, 19]. In the selfing species, it was estimated that RI may have developed over just a few millennia, and these reproductively isolated populations cannot be distinguished morphologically. Such ‘cryptic species’ are found in selfing and outcrossing species, but raise the issue of species delimitation, which has been argued to be uneven between mating systems [20]. For instance, convergent evolution of floral traits in the context of selfing syndrome [21] may lead to selfing species that are morphologically similar but reproductively isolated. In contrast, because morphological variation among populations may be more discrete in selfing species compared to outcrossing species, populations of selfing species may be more likely considered as different taxonomic species even if they belong to a single biological species [20]. Therefore, using experimental crosses to test the effects of selfing on speciation requires both measurements of RI and estimates of genetic divergence between populations or species. Such data would allow to test whether RI evolves at different paces within selfing *vs*. outcrossing lineages.

Experimental crosses also allow to address the genetic architecture of RI. Quantitative trait loci analyses of F2 populations of the predominantly selfing species *Draba nivalis* showed that post-zygotic incompatibilities in this Arctic species are due to single-locus underdominance, a putative chromosomal translocation, and nuclear-nuclear and cyto-nuclear epistatic incompatibilities [22, 23].

Theoretical expectations on the effects of selfing on speciation are poorly studied and not straightforward. Selfing may affect speciation by excluding evolutionary forces such as sexual conflicts (*e.g*., conflict between parents for resource allocation to the developing embryos) and sexual selection (*e.g*., pollen and sperm competition for fertilisation) [24], which often are found to promote speciation [25–27]. Importantly, selfing may affect speciation by modifying key population genetics parameters. First, selfing decreases gene flow within and among populations, enhancing their isolation and thus possibly facilitating speciation [7]. Second, the non-random sampling of gametes used for reproduction by selfing individuals reduces the effective population size. For instance, the effective population size is expected to be halved in purely selfing species compared with a randomly mating outcrossing species with the same population size [28, 29]. A reduction of the effective population size has cascading effects. It elevates genetic drift, reduces genetic polymorphism, and overall weakens selection. Third, selfing increases homozygosity, which makes recombination less efficient because homologous chromosomes tend to be identical [29]. Thus, recombination breaks down linkage disequilibrium less efficiently in strongly selfing populations, reducing the evolutionary advantages of recombination [30], and overexposing the populations to the deleterious effects of linked selection, such as background selection [31], further reducing the effective population size [32, 33].

Here, we developed analytical and simulation models of population genetics to better understand the effects of selfing on speciation. We did not consider the effects of gene flow and potential heterosis, but focused on understanding how the interplay between genetic drift, genetic linkage, and selection efficacy affects the accumulation of hybrid incompatibility. We studied how mutations leading to genetic incompatibility between populations accumulate within populations that differ in selfing rates, and provide predictions on (i) the pace of accumulation of genetic incompatibilities and (ii) the genetic architecture of RI in selfing *vs*. outcrossing lineages.

We sequentially explored three types of mutations: underdominant mutations, compensatory mutations, and Bateson-Dobzhansky-Muller incompatibility mutations. Underdominant mutations have deleterious effects in heterozygotes, but have no deleterious effects in homozygotes, and may for instance be due to structural variants [34]. Compensatory mutations are a pair of mutations that are both deleterious when they occur alone in a genome, but neutral when they occur together [35] (*e.g*., compensatory evolution of *cis*- and *trans*-regulation of gene expression [36–38]). Bateson-Dobzhansky-Muller incompatibility (BDMi) mutations are a pair of mutations that have no deleterious effects when they occur alone in a genome, but cause genetic incompatibilities when they occur together [39–42]. Importantly, fixation of underdominant and compensatory mutations requires the population to pass through a fitness valley, where the mutations may be counter-selected. In contrast, fixation of BDMi mutations may be neutral or even positively selected [43]. Because homozygotes are formed more readily in selfing species, selfing has been shown to facilitate the fixation of underdominant mutations [44]. It is however unknown if and how selfing modulates the accumulation of mutations with epistatic effects, such as compensatory and BDMi mutations.

Overall, we hypothesised that the effects of selfing on the accumulation of genetic incompatibilities depend on the mode of speciation. If genetic incompatibilities arise through genetic drift, selfing should promote their accumulation because underdominant and compensatory mutations are more likely to get fixed through genetic drift. In contrast, if genetic incompatibilities arise as a by-product of selection (*e.g*., ecological speciation), we expect that they arise most readily in outcrossing lineages because selection is less efficient in selfing populations.

## Methods

Accumulation of BDMi in allopatry has mainly been modelled as a combinatorial process of substitutions, each predicted by single-locus theory [42, 43]. Extending such models to selfing populations would be straightforward but partly misleading, as they do not explicitly consider the underlying multi-locus population genetics dynamics and the possible interactions among alleles that can be affected by selfing. Instead, we studied the effects of selfing on speciation by modelling—in a single population—the fate of different types of mutations that create genetic incompatibilities among populations. We determined the probability of and the time to fixation. Fixation of a single incompatibility mutation is, in most cases, not sufficient to complete speciation but determines the overall pace at which hybrid incompatibility builds up.

We built analytical models of single-locus incompatibilities (*i.e*., underdominant mutations) and multi-locus incompatibilities (*i.e*., compensatory and BDMi mutations), aiming to predict the effects of selfing on the time to fixation. We then tested the analytical predictions by building two types of simulation models. First, we performed single-locus simulations (for the underdominant mutations) and two-locus simulations (for the compensatory and BDMi mutations) to characterize the underlying mechanisms by which mutations go to fixation. These models allowed us to further test the effects of background selection (see below). Second, to extend this to a genome scale, we performed multi-locus simulations in which mutations can occur recurrently throughout the genome.

### Analytical models

For all models, we considered a population of *N* hermaphroditic individuals reproducing by selfing at rate 0 ≤ *σ* ≤ 1 (Table 1). The effective size of partially selfing populations is given by Pollak [28]:

$$\begin{array}{c} N_{e}=\frac{N}{1+F} \end{array}$$ (1)

where *F*, the Wright’s fixation index, is:

$$\begin{array}{c} F=\frac{\sigma }{2-\sigma } \end{array}$$ (2)

Note that the effective population size, *N*_*e*_, can be further reduced in selfing populations due to background selection, which we included in the single-locus and two-locus simulations (see Simulations).

**Table 1:** Glossary of the main notations.

Symbol	Biological meaning
*N*	Population size
*N*_*e*_	Effective population size
*σ*	Selfing rate
*F*	Wright’s fixation index
*μ*	Mutation rate
*r*	Recombination rate
*L*	Genome length (used in multi-locus simulation models only)
*h*	Dominance coefficient (besides genetic incompatibilities)
*s*	Strength of selection (besides genetic incompatibilities)
*h*_*c*_, *h*_*b*_	Dominance coefficient of the compensatory and BDMi mutations
*s*_*u*_, *s*_*c*_, *s*_*b*_	Strength of selection of the underdominant, compensatory and BDMi mutations
*k*_*c*_, *k*_*b*_	Dominance coefficient in double heterozygotes for compensatory and BDMi mutations

We focused on the mean time to fix the first incompatibility allele or haplotype under recurrent mutations, which can be decomposed into the mean waiting time to occurrence of the first mutation destined to be fixed (*T*_*wait*_) and the mean fixation time conditioned on fixation (*T*_*fix*_):

$$\begin{array}{c} T=T_{wait}+T_{fix} \end{array}$$ (3)

**Fig 1:**
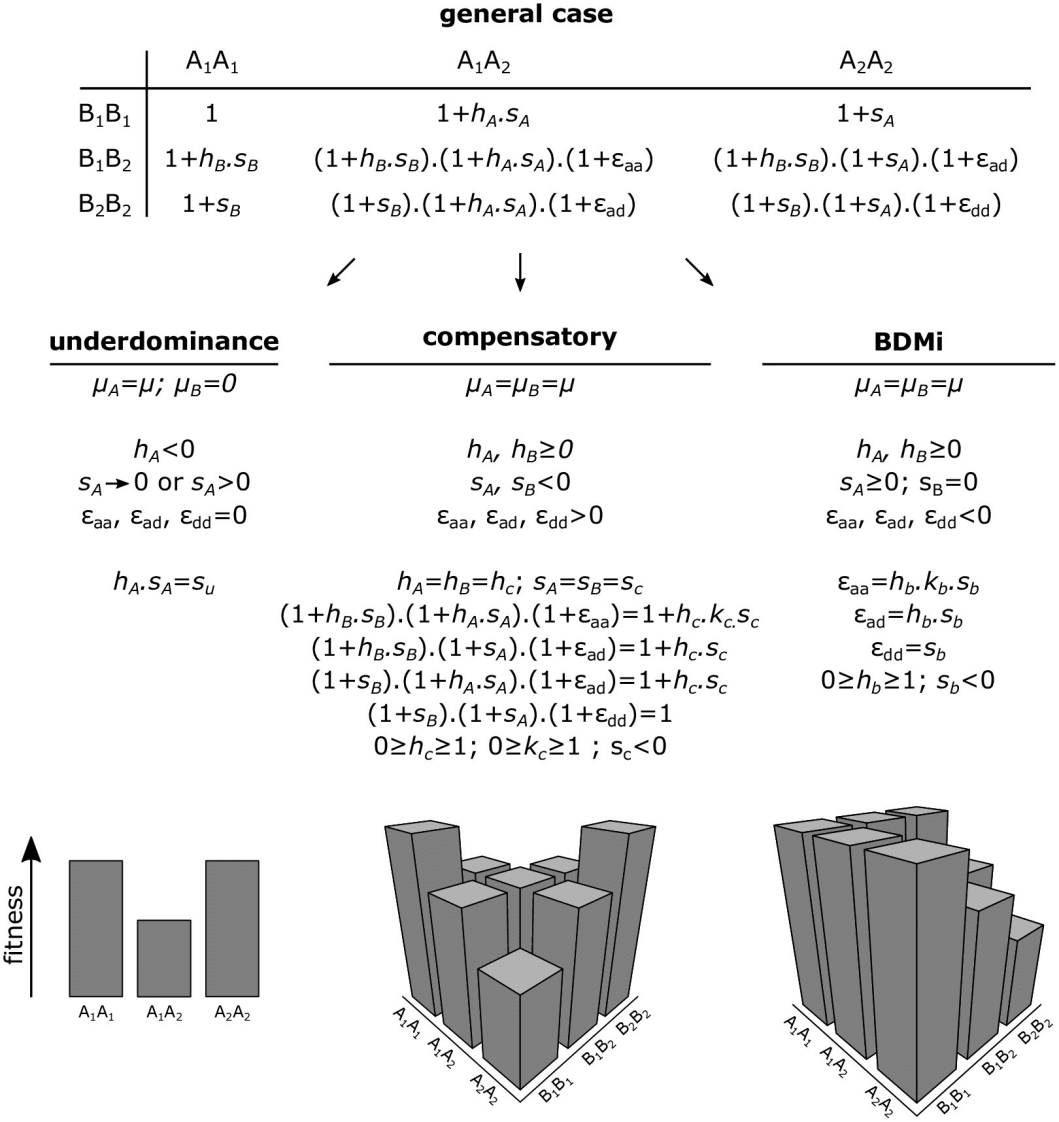
Fitness matrix of a symmetrical general case made of two biallelic loci with dominance coefficients (*h*_*A*_ and *h*_*B*_), selection coefficients (*s*_*A*_ and *s*_*B*_), and epistasis coefficients (*ϵ*_*aa*_, *ϵ*_*ad*_ and *ϵ*_*dd*_). The combinations of value ranges for these parameters explain our three models of genetic incompatibility. The 2D or 3D fitness landscapes represent unique combinations of parameters.

The different forms of incompatibilities can be encapsulated within a single general two-locus model with epistatic interactions as summarized in Fig 1. This illustrates the unity and connections among the different models but to avoid manipulating too many parameters, which may be not relevant for each specific case, we re-parametrised the models with some composite parameters that simplify equations and have clearer biological interpretations (Fig 1 and details below).

#### Single-locus incompatibility

The model of underdominant mutations has already been studied by Charlesworth [44] but we summarize it for the sake of completeness and provide additional results. We considered a single bi-allelic locus, with the ancestral allele *A*_1_ that can mutate to the derived allele *A*_2_ at rate *μ*. Following Fig 1, the fitness of genotypes *A*_1_*A*_1_, *A*_1_*A*_2_, and *A*_2_*A*_2_ are, 1, 1 + *h*_*A*_*s*_*A*_, and 1 + *s*_*A*_ with *h*_*A*_ < 0, respectively, and the frequency of allele *A*_2_ is noted *x*. To re-parametrised fitness as in standard underdominance models we set *s*_*u*_ = −*h*_*A*_*s*_*A*_ and remove the *A* superscript, so the three fitness are: 1, 1 − *s*_*u*_, and 1 + *s*.

The change in allele frequencies in one generation is given by:

$$\begin{array}{cc} \Delta x & =x\left(1-x\right)(\left(1-F\right)(2\left(2s_{u}+s\right)x-2s_{u})+Fs)/\bar{W}\\ & \approx x\left(1-x\right)(\left(1-F\right)(2\left(2s_{u}+s\right)x-2s_{u})+Fs) \end{array}$$ (4)

where $\bar{W}$ is the mean fitness of the population, approximately equal to 1 when selection is weak. Eq (4) can also be written as:

$$\begin{array}{cc} \Delta x & \approx 2\left(1-F\right)\left(2s_{u}+s\right)x\left(1-x\right)\left(x-x_{eq}\right)\;\text{if}\;F<1\\ & \approx sx(1-x)\;\text{if}\;F=1 \end{array}$$ (5)

with

$$\begin{array}{c} x_{eq}=\frac{2s_{u}-F\left(2s_{u}+s\right)}{2\left(1-F\right)\left(2s_{u}+s\right)} \end{array}$$

According to diffusion theory, the probability of fixation of a single *A*_2_ mutant is given by:

$$\begin{array}{c} P_{fix}=\frac{\int_{0}^{1/2N}\text{exp}\left(-\frac{2M_{\delta x}}{V_{\delta x}}\right)dx}{\int_{0}^{1}\text{exp}\left(-\frac{2M_{\delta x}}{V_{\delta x}}\right)dx} \end{array}$$ (6)

where *M*_*δx*_ = Δ*x* is the expected infinitesimal change in allele frequency, and $V_{\delta x}=\frac{x(1-x)}{2N_{e}}$ is the expected infinitesimal variance.

#### Two-locus incompatibilities

We also considered models with two bi-allelic loci. *A*_1_ and *A*_2_, *B*_1_ and *B*_2_ denote the ancestral and derived alleles at the first and second locus, respectively, with frequencies *x* and 1 − *x*, *y* and 1 − *y*. We assumed the same mutation rate, *μ*, at the two loci. The recombination rate between the two loci is 0 ≤ *r* ≤ 0.5, where 0 corresponds to fully linked loci and 0.5 corresponds to independent loci, for example located on different chromosomes. The frequency of the four haplotypes {*A*_1_*B*_1_, *A*_1_*B*_2_, *A*_2_*B*_1_, *A*_2_*B*_2_} are denoted as {*X*_1_, *X*_2_, *X*_3_, *X*_4_}, and the frequency of the ten genotypes, the combination of haplotypes *X*_*i*_ and *X*_*j*_, are denoted as *G*_*ij*_ with *i* ≤ *j* (for example *G*_12_ = [*A*_1_*A*_1_;*B*_1_*B*_2_]). Note that we must distinguish *G*_14_ from *G*_23_, which correspond to identical genotypes [*A*_1_*A*_2_;*B*_1_*B*_2_] but may differ in the haplotypes produced through gametogenesis. Changes in genotype frequencies can be obtained as follows [44]:

After meiosis, haplotype frequencies are given by:

$$\begin{array}{cc} X_{1}^{\prime } & =G_{11}+\frac{1}{2}\left(G_{12}+G_{13}+\left(1-r\right)G_{14}+rG_{23}\right)\\ X_{2}^{\prime } & =G_{22}+\frac{1}{2}\left(G_{12}+G_{24}+\left(1-r\right)G_{23}+rG_{14}\right)\\ X_{3}^{\prime } & =G_{33}+\frac{1}{2}\left(G_{13}+G_{34}+\left(1-r\right)G_{23}+rG_{14}\right)\\ X_{4}^{\prime } & =G_{44}+\frac{1}{2}\left(G_{24}+G_{34}+\left(1-r\right)G_{14}+rG_{23}\right) \end{array}$$

After syngamy, genotypic frequencies are given by:

$$\begin{array}{cc} G_{11}^{\prime } & =\left(1-\sigma \right)X_{1}^{\prime 2}+\sigma (G_{11}+\frac{1}{4}\left(G_{12}+G_{13}+r^{2}G_{23}+{\left(1-r\right)}^{2}G_{14}\right))\\ G_{22}^{\prime } & =\left(1-\sigma \right)X_{2}^{\prime 2}+\sigma (G_{22}+\frac{1}{4}\left(G_{12}+G_{24}+r^{2}G_{14}+{\left(1-r\right)}^{2}G_{23}\right))\\ G_{33}^{\prime } & =\left(1-\sigma \right)X_{3}^{\prime 2}+\sigma (G_{33}+\frac{1}{4}\left(G_{13}+G_{34}+r^{2}G_{14}+{\left(1-r\right)}^{2}G_{23}\right))\\ G_{44}^{\prime } & =\left(1-\sigma \right)X_{4}^{\prime 2}+\sigma (G_{44}+\frac{1}{4}\left(G_{24}+G_{34}+r^{2}G_{23}+{\left(1-r\right)}^{2}G_{14}\right))\\ G_{14}^{\prime } & =\left(1-\sigma \right)2X_{1}^{\prime }X_{4}^{\prime }+\sigma \frac{1}{2}\left(r^{2}G_{23}+{\left(1-r\right)}^{2}G_{14}\right)\\ G_{23}^{\prime } & =\left(1-\sigma \right)2X_{2}^{\prime }X_{3}^{\prime }+\sigma \frac{1}{2}\left(r^{2}G_{14}+{\left(1-r\right)}^{2}G_{23}\right)\\ G_{ij}^{\prime } & =\left(1-\sigma \right)2X_{2}^{\prime }X_{3}^{\prime }+\sigma \frac{1}{2}\left(G_{ij}+r\left(1-r\right)\left(G_{14}+G_{23}\right)\right)\;\text{for}\;\text{other}\;\text{cases} \end{array}$$

Finally, after selection:

$$\begin{array}{c} G_{ij}^{sel}=\frac{w_{ij}G_{ij}^{\prime }}{\sum_{i\leq j}w_{ij}G_{ij}^{\prime }} \end{array}$$ (7)

where *w*_*ij*_ is the fitness of genotype *G*_*ij*_, which depends on the type of two-locus incompatibility studied (see compensatory mutations and BDMi mutations below). Note that in either scenario, we do not distinguish *cis* and *trans* effects on fitness (*i.e*., *w*_14_ = *w*_23_).

We extended previous models of compensatory mutations [45, 46] by including the effects of partial selfing. Compensatory mutations at two loci can be viewed as a generalization of the one-locus underdominant model presented above where two haplotypes are equally fit, *A*_1_*B*_1_ and *A*_2_*B*_2_, but the intermediate paths, *A*_1_*B*_2_ and *A*_2_*B*_1_ are deleterious. Thus, as for underdominant mutations, the evolution of pairs of compensatory mutations requires crossing of a fitness valley. We consider a symmetrical fitness landscape where initial mutation are deleterious so *s*_*A*_ = *s*_*B*_ = −*s*_*c*_ and *h*_*A*_ = *h*_*B*_ = *h*_*c*_ (the *c* stands for compensatory). To simplify the notation, we summarized epistatic effects such that genotypes have the following fitness (see Fig 1):

$$\begin{array}{cc} w_{11}=w_{44} & =1\\ w_{22}=w_{33} & =1-s_{c}\\ w_{12}=w_{13}=w_{24}=w_{34} & =1-h_{c}s_{c}\\ w_{14}=w_{23} & =1-h_{c}k_{c}s_{c} \end{array}$$

where *s*_*c*_ ≥ 0 and 0 ≤ *h*_*c*_ ≤ 1 are the strength and the dominance coefficient of the deleterious effects of each mutation respectively, and *k*_*c*_ is the dominance coefficient for the double heterozygous genotype *A*_1_*A*_2_*B*_1_*B*_2_.

We developed a model of BDMi mutations by incorporating the effects of partial selfing into the model of Kimura and King [47]. BDMi mutations generate genetic incompatibility only when an individual carries both derived alleles. Thus, individuals carrying either derived allele do not experience deleterious effects, meaning that the fixation of BDMi mutations does not require crossing of a fitness valley. We assumed either a purely neutral BDMi model, that is *s*_*A*_ = *s*_*B*_ = 0, or a BDMi model with one of the locus involved in local adaptation, say *s*_*A*_ > 0. Implicitly, positive selection at the *A* locus occurs in the focal population that is modelled but not in the other diverging population, where selection could occur on the other locus (so *s*_*A*_ > 0 and *s*_*B*_ = 0 in one population and *s*_*B*_ > 0 and *s*_*A*_ = 0 in the other). To lighten notations, we set *s*_*A*_ = *s* and *h*_*A*_ = *h* and wrote epistasis coefficients as a function of the selection coefficient against the two derived alleles *A*_2_ and *B*_2_, *s*_*b*_ (*b* standing for BDMi) and two dominance coefficients, *h*_*b*_ for single heterozygotes, and *k*_*b*_*h*_*b*_ for double heterozygotes. The fitness of the genotypes are as follows:

$$\begin{array}{cc} w_{11}=w_{12}=w_{22} & =1\\ w_{13} & =1+hs\\ w_{33} & =1+s\\ w_{24} & =\left(1+hs\right)\left(1-h_{b}s_{b}\right)\\ w_{34} & =\left(1+s\right)\left(1-h_{b}s_{b}\right)\\ w_{14}=w_{23} & =\left(1+hs\right)\left(1-h_{b}k_{b}s_{b}\right)\\ w_{44} & =\left(1+s\right)\left(1-s_{b}\right) \end{array}$$

Finally, we also considered convergent (and symmetrical) selection where both loci can contribute to adaptation: *s*_*A*_ = *s*_*B*_ = *s* > 0 (see [43] for a classification of the different cases presented here). Under this scenario, the fitnesses are:

$$\begin{array}{cc} w_{11} & =1\\ w_{12}=w_{13} & =1+hs\\ w_{22}=w_{33} & =1+s\\ w_{24}=w_{34} & =\left(1+hs\right)\left(1+s\right)\left(1-h_{b}s_{b}\right)\\ w_{14}=w_{23} & ={\left(1+hs\right)}^{2}\left(1-h_{b}k_{b}s_{b}\right)\\ w_{44} & ={\left(1+s\right)}^{2}\left(1-s_{b}\right) \end{array}$$

### Simulations

We simulated all three models using single-locus, two-locus, and multi-locus models to check our analytical and numerical predictions.

#### Single-locus and two-locus simulations

We developed programs to simulate populations of *N* hermaphroditic individuals producing gametes with mutations from ancestral to derived alleles at a rate *μ* and, for the two-locus simulations, with recombination events between the two loci at a rate 0 ≤ *r* ≤ 0.5. Self-fertilisation may occur at a rate 0 ≤ *σ* ≤ 1. Then, selection on offspring occurs differently in the underdominant, compensatory, and BDMi mutations simulations (as described above). Finally, genetic drift was included by sampling offspring from the genotype frequencies using a multinomial distribution using the *gsl*_*ran*_*multinomial* function from the GNU Scientific Library [48]. For each iteration, we measured the number of generations required to fixate the derived allele (underdominant mutation), a pair of derived alleles (compensatory mutations), or either derived allele (BDMi mutations).

We included the effects of background selection in our single-locus and two-locus simulation models. Selfing is known to increase the strength of background selection, which further reduces effective population size [32, 33, 49]. The strength of this effect critically depends on genomic recombination rate. When the genomic recombination rate is high, only highly selfing populations suffer from background selection. When the genomic recombination rate is low, the effect of background selection rises linearly with selfing [32, 33]. To account for this effect, we modelled background selection as a simple additional reduction of *N*_*e*_. We used a Dirichlet-multinomial distribution in the genetic drift function (instead of a multinomial distribution) that allowed us to tailor the effective size of the population to its selfing rate, independently of census population size, *N*. The Dirichlet-Multinomial distribution is a multinomial distribution where the vector of probability is itself drawn in a Dirichlet distribution of parameter *α*. Compared to a multinomial distribution, the variance of genotypic frequency changes is inflated by a factor $\frac{N+\alpha }{1+\alpha }$, so that:

$$\begin{array}{c} N_{e}=\frac{1+\alpha }{N+\alpha }N \end{array}$$ (8)

We used analytical approximations in Roze [33] to determine *N*_*e*_ under two background selection scenarios, corresponding to two levels of genomic recombination. The first scenario corresponds to a low rate of genomic recombination and leads to a linear decrease in *N*_*e*_ with selfing rate (hereafter ‘linear BG effects’). The second scenario corresponds to a high rate of genomic recombination and leads to an accelerating decrease in *N*_*e*_ with selfing rate (hereafter ‘curved BG effects’). Specifically, using the BS1 function from the supplemental material of Roze [33] (File S2), we modelled the background selection effects assuming that deleterious alleles with selection and dominance coefficients of *s* = 0.05 and *h* = 0.2 occurred with a genomic mutation rate of *U* = 0.1 in a genome map length of *R* = 0.5 (’linear BG effects’) or *R* = 40 (’curved BG effects’). This informed us about by how much *N*_*e*_ was reduced due to background selection for different selfing rates. Then, we used the predicted *N*_*e*_ to compute the corresponding Dirichlet parameter *α* to use in the simulation, simply by inverting Eq (8).

#### Multi-locus simulations

We used individual based forward simulations in which the individuals have diploid genomes of length *L* elements representing loci on which mutations can occur at rate *μ* and between which recombination can occur at rate *r*. We modelled all the types of genetic incompatibility outlined above.

The single-locus and two-locus simulations were performed using C++ scripts, and the multi-locus simulations were performed with the software SLiM [50], both using GNU parallel [51] (see S1 Scripts).

## Results

### Underdominant mutations

Our analytical and simulation models allowed us (i) to study the effects of *s*_*u*_ and *s* on the accumulation of underdominant mutations; (ii) to test how background selection alters this dynamic; and (iii) to develop simple analytical predictions when the strength of the selection coefficient (*s*_*u*_) is not fixed but drawn from a distribution of fitness effects.

#### Effects of *s*_*u*_ and *s* on underdominant mutations

Underdominant mutations arising in a population are first counter-selected, and it is well known that either genetic drift or selfing facilitates crossing the fitness valley [44]. For the sake of completeness, we first summarize previous results (S1 Fig and S1 Appendix). With higher selfing and lower heterozygote frequencies, selection against underdominant mutations becomes less important, increasing their fixation probability and decreasing their time to fixation. A new mutation may be positively selected if the population’s selfing rate is high enough, that is if:

$$\begin{array}{c} \sigma >\sigma_{lim}=\frac{2s_{u}}{s+2s_{u}} \end{array}$$ (9)

#### Effects of background selection on underdominant mutations

Including the effects of background selection increases the time to fixation when the homozygotes *A*_2_*A*_2_ are positively selected (*s* > 0) in highly selfing populations (S1(E) Fig). This is because background selection reduces the effective population size, *N*_*e*_, and thereby the efficacy of selection. Such a low efficacy of selection reduces both the fitness valley in the heterozygotes, *A*_1_*A*_2_ and the fitness peak in the homozygotes, *A*_2_*A*_2_. However, because background selection mostly impacts highly selfing populations, in which selection on heterozygotes is irrelevant, the effect of background selection mostly manifests as a lower probability of fixation in highly selfing populations.

#### Distribution of the underdominant effect on fitness

**Fig 2:**
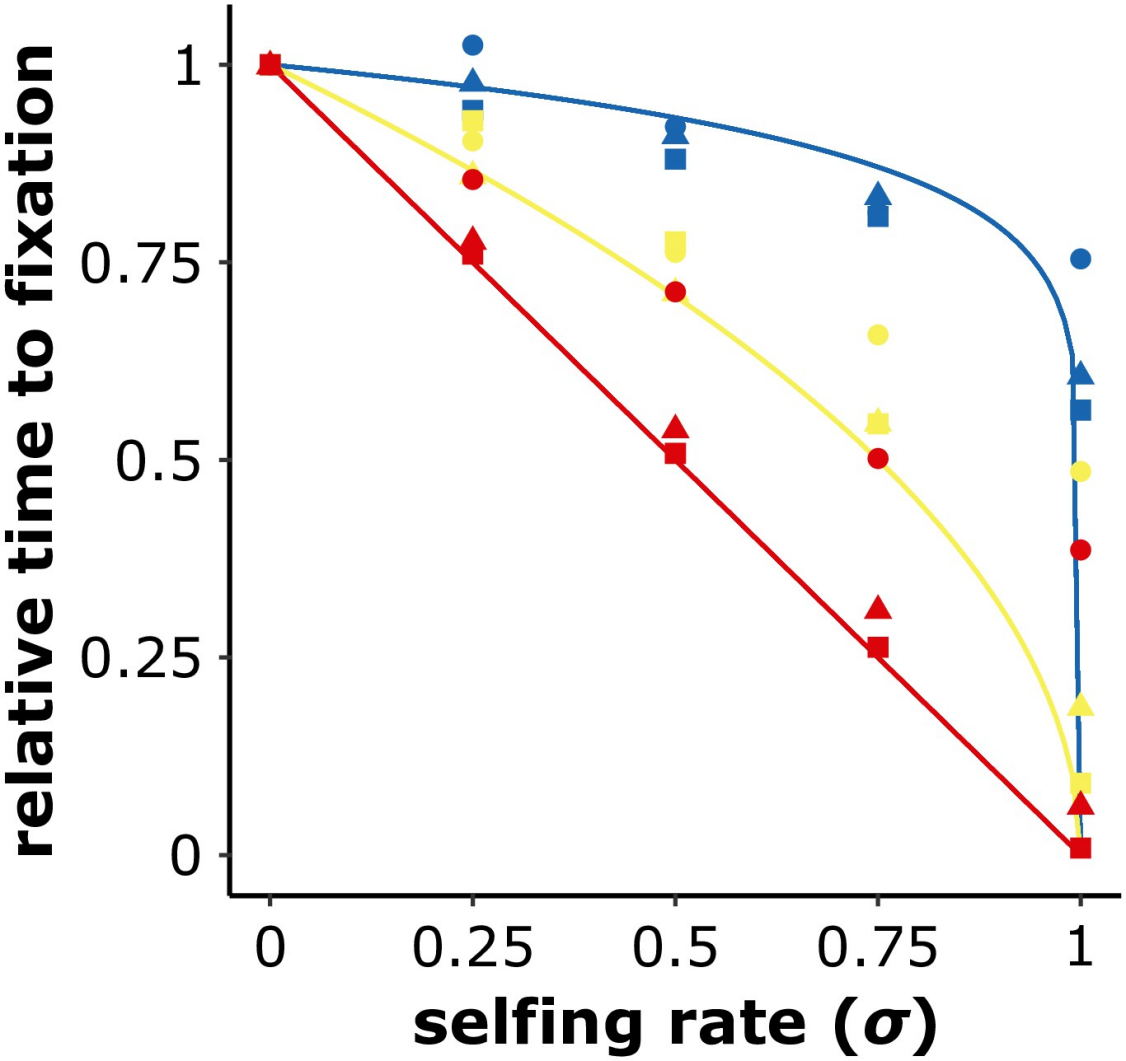
Underdominant mutations accumulate more rapidly in selfing populations. Analytical predictions (lines) and outcomes of multi-locus simulations (symbols) of the averaged number of generations required to fixate an underdominant mutation compared to an outcrossing population (*σ* = 0). 4*Ns*_*u*_ follows a Gamma distribution with a shape parameter *β* of 0.1 (blue), 0.5 (yellow), or 1 (red), and mean parameter *γ* of 10 (circles), 100 (triangles), or 1,000 (squares). *L* = 100, *N* = 1, 000, *μ* = 10^−6^, *r* = 0.01. The analytical predictions are (1 − *σ*)^*β*^. 1, 000 iterations.

Previous studies have only considered a single mutation with constant effect [44]. However, if we consider a flow of underdominant mutations, it is more realistic that their fitness effects vary. For example, small chromosome inversions (which can generate underdominance) are more likely to have a smaller effect than bigger ones [52]. As is often assumed for deleterious mutations [53] and to gain sufficient flexibility, we assumed that 4*Ns*_*u*_ follows a Gamma distribution with mean *γ* and shape *β*. We found that the proportion of underdominant mutations that go to fixation in partially selfing populations compared to outcrossing populations is well approximated by:

$$\begin{array}{c} {\left(1-\sigma \right)}^{-\beta } \end{array}$$ (10)

which is independent of *γ*. Hence, because the time to fixation is mostly determined by the waiting time for the mutation that will be fixed to arise, the time to fixation in partially selfing compared to outcrossing populations is given by:

$$\begin{array}{c} {\left(1-\sigma \right)}^{\beta } \end{array}$$ (11)

which our multi-locus simulation results confirm as long as *γ* is not too small (Fig 2). It shows that when *β* < 1 there is a non-linear accelerating effect of selfing on the accumulation of underdominant mutations, and the lower *β* is the stronger the effect.

### Compensatory mutations

We found that the strength of the deleterious effects of compensatory mutations (*s*_*c*_) affects how fixation takes place in outcrossing populations. We derived analytical predictions, and confirmed them with two-locus and multi-locus simulations, when selection is (i) weak or (ii) strong, before (iii) incorporating the effects of background selection.

#### Compensatory mutations with weak effects

How selfing affects the fixation of mutations depends first on the strength of the deleterious effect relative to neutrality (*N*_*e*_*s*_*c*_ of the order of 1 or lower). When the deleterious effect is small, fixation occurs in two steps. A first weakly deleterious mutation goes to fixation and then a second, compensatory, mutation restoring fitness goes to fixation, behaving as weakly beneficial. The total fixation time can thus be approximated by:

$$\begin{array}{c} T_{weak}\approx T_{deleterious}+T_{beneficial} \end{array}$$ (12)

It is important to note that dominance of the first and second mutations is reverse. The compensatory mutation arises in a genetic background of fitness 1 − *s*_*c*_, so rescaling the fitness of heterozygote and homozygote genotypes for the compensatory mutation leads to the following relative fitness: 1 − *h*_*c*_*s*_*c*_/1 − *s*_*c*_ ≈ 1 + (1 − *h*_*c*_)*s*_*c*_ and 1/1 − *s*_*c*_ ≈ 1 + *s*_*c*_, respectively. The effect of selfing thus depends on the dominance coefficient (*h*_*c*_), and the fixation is either faster or slower in selfing populations than in outcrossing populations. Selfing speeds up the fixation of recessive beneficial and dominant deleterious mutations [54]. So, the fixation of compensatory mutations is faster under selfing when *h*_*c*_ > 1/2 whereas it is faster under outcrossing when *h*_*c*_ < 1/2 (S2 Fig).

#### Compensatory mutations with strong effects

**Fig 3:**
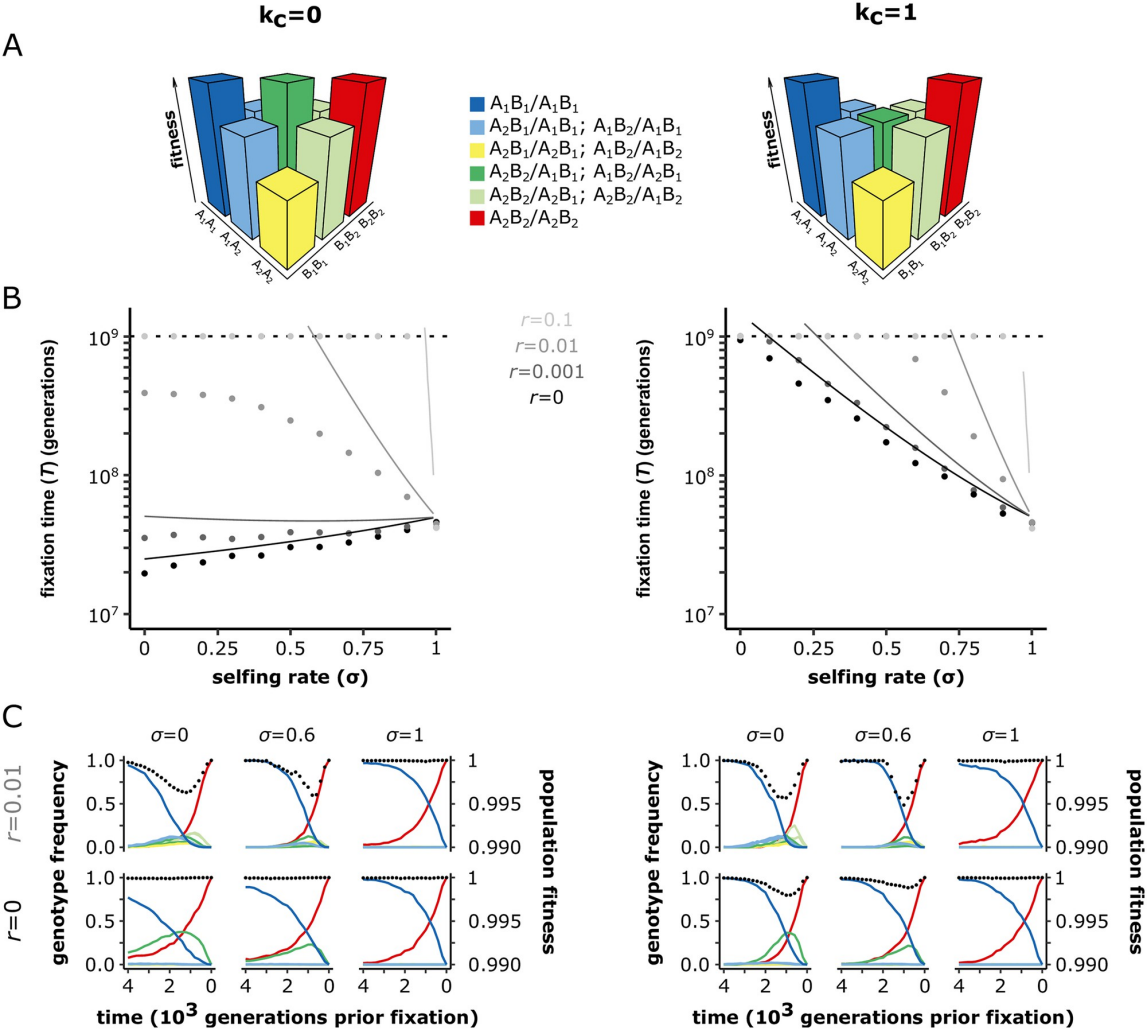
The effect of selfing on the fixation of compensatory mutations depends on the dominance coefficient in the double heterozygote (*k*_*c*_), and the recombination rate(*r*). (A) Schematic representations of the fitness landscapes for compensatory mutations with *k*_*c*_ = 0 (left) or *k*_*c*_ = 1 (right). *A*_1_ and *B*_1_ are the ancestral alleles. *A*_2_ and *B*_2_ are the derived alleles. (B) Time to fixation of a pair compensatory mutations with *k*_*c*_ = 0 (left) or *k*_*c*_ = 1 (right). The lines correspond to the analytical approximations (Eq 14) for *r* = 0 (black), *r* = 0.001 (dark grey), *r* = 0.01 (grey) and *r* = 0.1 (light grey). Dots show the corrected mean times to fixation of a pair of compensatory mutations from our two-locus simulations. We corrected the raw means because the threshold made our data right censored (*i.e*., missing estimates above 10^9^ generations), which we accounted for by first estimating the full distribution by fitting gamma distributions on our simulation outputs using the *fitdistriplus* R package [55], from which we estimated the corrected mean times to fixation. The dashed horizontal lines indicate the generation threshold after which simulations stop. *N* = 1, 000, *μ* = 10^−5^, *h*_*c*_ = 0.5, *s*_*c*_ = 0.01. 1, 000 *iterations*. (C) Population fitness (black dots—right y axis) and the frequencies of the 10 possible genotypes on the two loci fitness landscapes (solid lines—left y axis) over the last 4200 generations preceding the fixation of the pair of compensatory mutations. The line colours match the genotype colours on the fitness landscapes. *N* = 1, 000, *μ* = 10^−5^, *h*_*c*_ = 0.5, *s*_*c*_ = 0.01. 100 iterations. See S3 and S4 Figs for the visualisation of additional parameter combinations.

**Fig 4:**
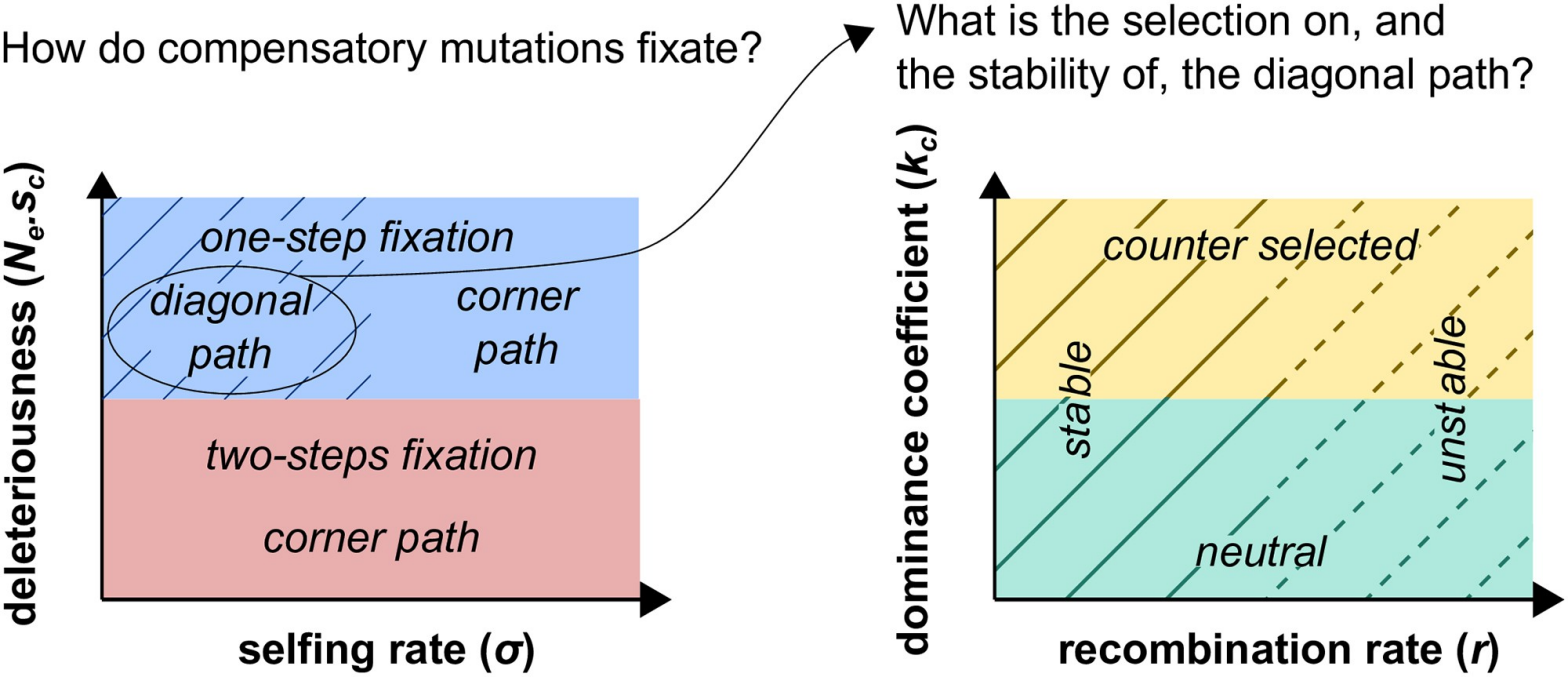
Fixation regime of compensatory mutations as a function of the selfing rate (*σ*), the strength of deleterious effect (deleteriousness), the recombination rate between the two loci (*r*), and the dominance coefficient in double heterozygotes (*k*_*c*_). Populations with high selfing rates always take the corner path on the fitness landscape (*i.e*., the deepest point of the fitness valley represented by genotypes that are homozygous for the ancestral allele at one locus, and homozygous for the derived allele at the other locus), while populations with low selfing rates can take the diagonal path (*i.e*., double heterozygous genotype). The diagonal path may however be counter selected (when *k*_*c*_ is relatively high) and unstable (when the recombination rate is relatively high). Note that, for the sake of simplicity, all effects are here presented as discrete categories while they are in fact gradual.

When the deleterious effect is too high to allow the fixation of a single mutation (*N*_*e*_*s*_*c*_ > > 1), the two compensatory mutations must segregate together in the population and go to fixation together. In this case, the key parameters are the recombination rate between loci (*r*), and the dominance coefficient in the double heterozygote (*k*_*c*_). When *r* = 0 and *k*_*c*_ = 0, we found that the fixation time of a pair of compensatory mutations (*T*_0,0_) may be approximated by (see S1 Appendix):

$$\begin{array}{c} T_{0,0}\approx \frac{\left(h_{c}\left(1-F\right)+F\right)s_{c}}{2\mu^{2}} \end{array}$$ (13)

which shows that selfing always increases the fixation time (Figs 3B and 4, and S3 Fig). This is because evolution can follow the diagonal path of the fitness landscape (*i.e*., through the double heterozygous genotype *A*_1_*A*_2_*B*_1_*B*_2_), and thus avoid the corner path made of genotypes with low fitness (*i.e*., *A*_1_*A*_1_*B*_2_*B*_2_ or *A*_2_*A*_2_*B*_1_*B*_1_), and this is easier in outcrossing than in selfing populations (Fig 3 and S4 Fig). In comparison to the corner path, the diagonal path represents a lower fitness valley, which flattens out as *k* approaches 0. Thus, when the diagonal path is neutral (*i.e*., *k*_*c*_ = 0) and recombination cannot occur between the two loci (*i.e*., *r* = 0), outcrossing populations fix compensatory mutations by forming the double mutated haplotype (*A*_2_*B*_2_). This is stable because it is not broken down by recombination and can spread in the population because it is not counter-selected.

When *k*_*c*_ > 0 and/or *r* > 0, the dynamics are very different. Either selection against double heterozygotes or recombination that breaks down double-mutated haplotypes considerably reduces the probability of crossing the fitness valley. When *r* is small, we can show that:

$$\begin{array}{c} T_{r,k}\approx T_{0,0}\frac{\sqrt{\pi }e^{R}Erf\left(\sqrt{R}\right)}{2\sqrt{R}} \end{array}$$ (14)

where *Erf* is the error function and *R* = *N*(1 − *σ*)(*r* + *hks*). The right-hand function increases very rapidly with *R*. These approximations and our simulations show that both recombination and selection against heterozygotes strongly impede the fixation of compensatory mutations, and that both effects are reduced by selfing (Figs 3B and 4, and S3 Fig). When *k*_*c*_ > 0, the diagonal path represents a fitness valley that selects against the double mutated haplotype (*A*_2_*B*_2_). And, when *r* > 0, the double mutated haplotype (*A*_2_*B*_2_) may break, forming genotypes that are off the diagonal path and eliminating the derived alleles (*A*_2_ and *B*_2_) (Figs 3 and 4). In contrast, the fixation time in highly selfing populations is less influenced by *r* and *k*_*c*_ because their high level of homozygosity (i) makes selfing populations not following the diagonal path of the fitness landscape, and (ii) makes the double mutated haplotype (*A*_2_*B*_2_) more stable over time due to the inefficient recombination in selfers [29].

Our multi-locus simulations confirmed that *r* and *k*_*c*_ critically affect the fixation time of compensatory mutations with strongly deleterious effects (S6 Fig).

#### Effects of background selection on compensatory mutations

Regardless of the dominance coefficient in the double heterozygote (*k*_*c*_) and the recombination rate (*r*), background selection shortens fixation time in highly selfing populations (S5 Fig). By increasing drift, background selection reduces the efficacy of selection against the deleterious mutation, which may thus segregate at higher frequency and be more likely to be associated with the compensatory mutation. Because of background selection, *N*_*e*_*s* can be small enough under selfing for fixation to occur in two steps (which is rapid see (13)) whereas, for the same population size, *N*_*e*_*s* can be too high under outcrossing so that fixation can only occur in a single step (which takes much longer (14)).

### BDMi mutations

**Fig 5:**
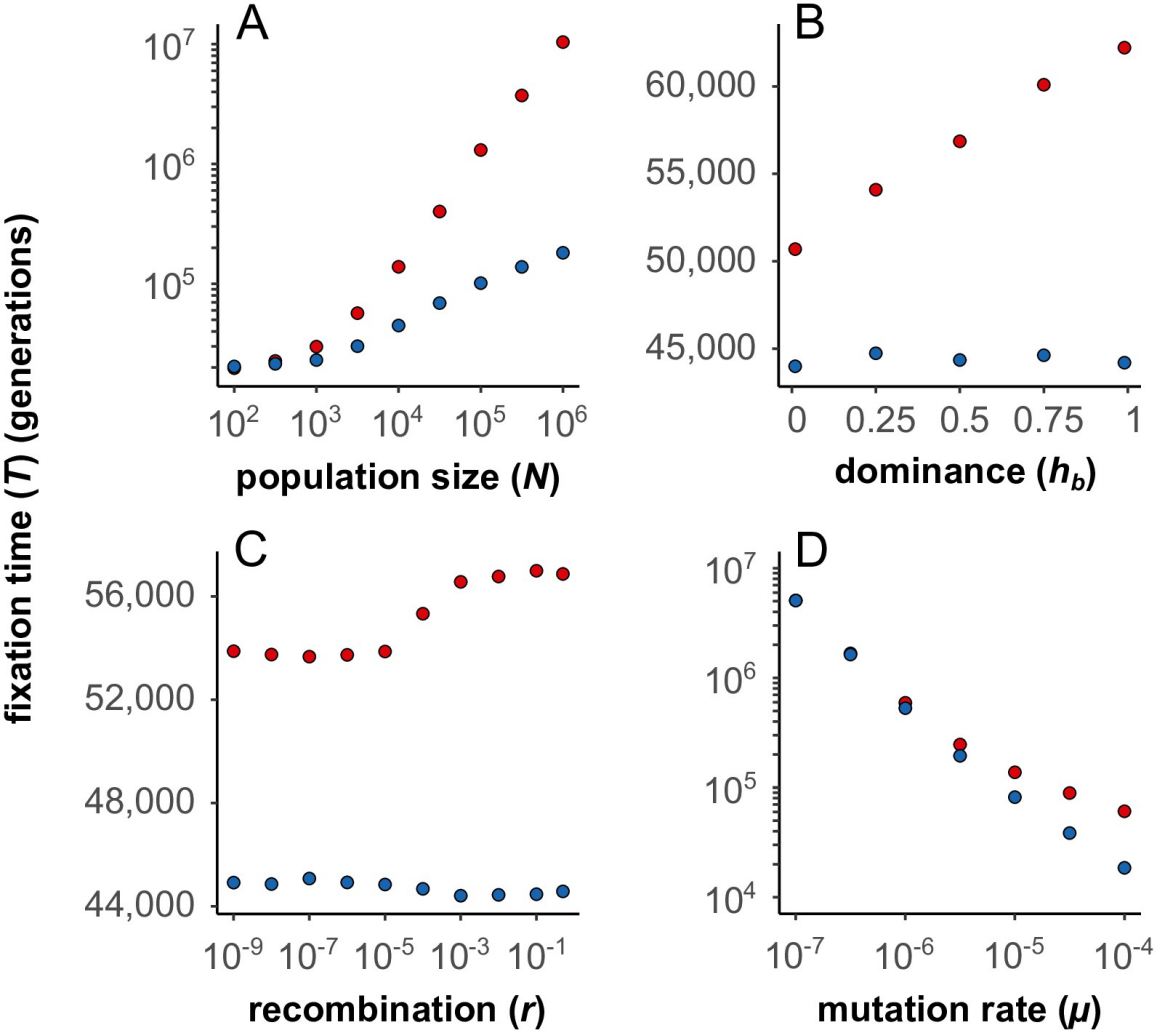
Fixation time of BDMi mutations (red) compared to neutral mutations (blue) in outcrossing populations (*σ* = 0) (two-locus model). (A) Higher population sizes (N) decelerate the fixation of BDMi mutations (*s*_*B*_ = 2.5.10^−2^, *h*_*B*_ = 0.5; red) compared to neutral mutations (*s*_*b*_ = 0; blue). *r* = 0.5, *μ* = 2.5.10^−5^. 1, 000 iterations. (B) Higher coefficients of dominance (*h*_*b*_) decelerate the fixation of BDMi mutations (*s*_*b*_ = 2.5.10^−4^; red) compared to neutral mutations (*s*_*b*_ = 0; blue). *N* = 10, 000, *r* = 0.5, *μ* = 2.5.10^−5^. 10, 000 iterations. (C) Higher rates of recombination (*r*) decelerate the fixation of BDMi mutations (*s*_*b*_ = 2.5.10^−4^, *h*_*b*_ = 0.5; red) compared to neutral mutations (*s*_*b*_ = 0; blue). *N* = 10, 000, *μ* = 2.5.10^−5^. 100, 000 iterations. (D) Higher rates of mutation (*μ*) decelerate the fixation of BDMi mutations (*s*_*b*_ = 2.5.10^−3^, *h*_*b*_ = 0.5; red) compared to neutral mutations (*s*_*b*_ = 0; blue). *N* = 10, 000, *r* = 0.5. 10, 000 iterations.

We were not able to find an analytical solution or approximation for the fixation time of mutations with BDMi mutations. Therefore, (i) we compared our simulations with neutral expectations to understand how genetic incompatibilities affect the fixation of mutations within populations (Fig 5), and how this dynamic differs in selfing populations. Moreover, we (ii) studied the effects background selection, and (iii) explored scenarios where selection decreases with selfing.

#### Analytical predictions for neutral mutations (*s* = 0) without incompatibilities

For neutral BDMi mutations (*s* = 0), when the mutation rate is low compared to neutrality (4*N*_*e*_*μ* < 1), the two incompatible alleles (*A*_2_ and *B*_2_) rarely segregate at the same time in the population, and one can consider that they go to fixation independently. According to basic results of the neutral theory, Eq 3 reduces to:

$$\begin{array}{c} T=\frac{1}{2\mu }+4N_{e} \end{array}$$ (15)

**Fig 6:**
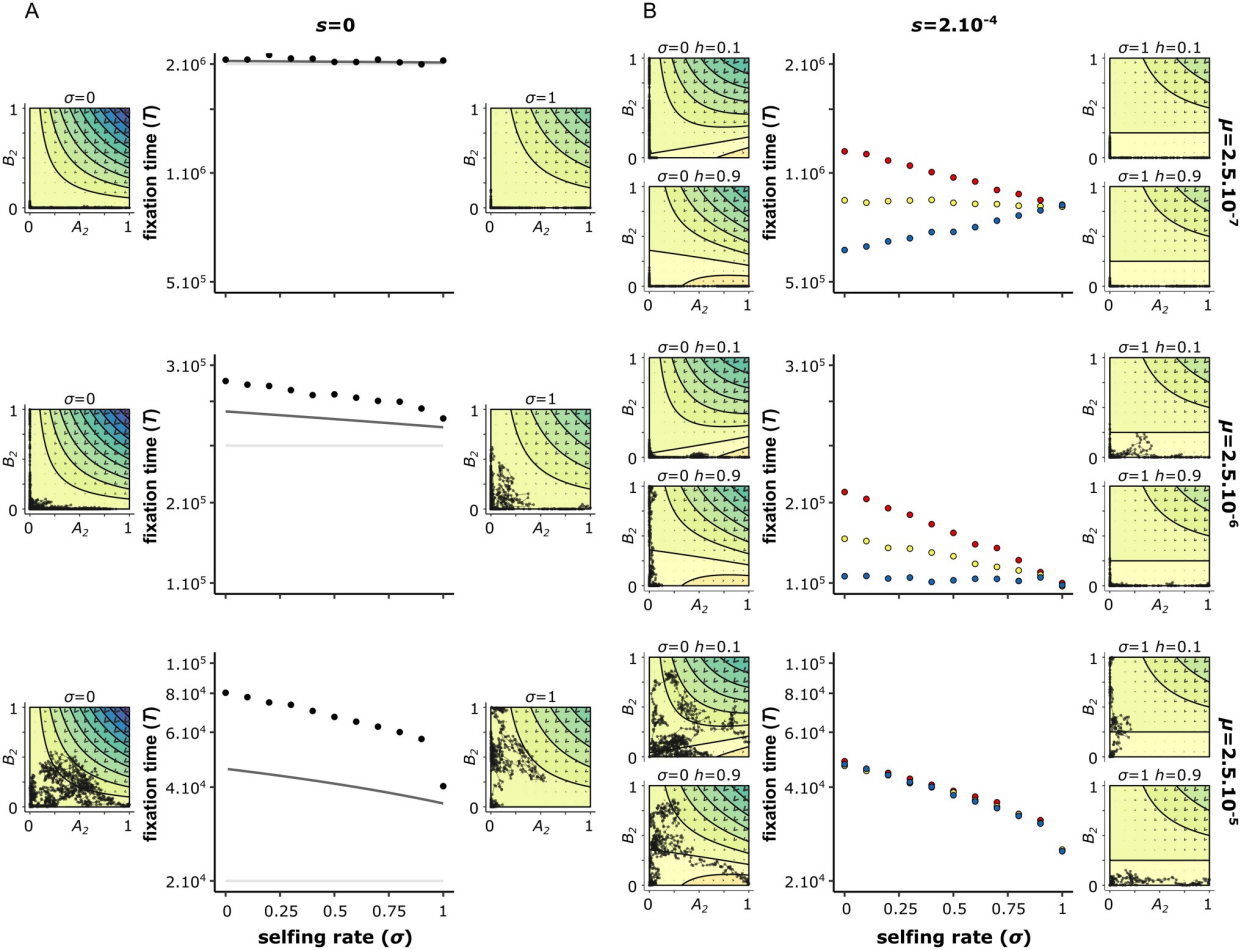
Selfing rate accelerates the fixation of BDMi mutations for high mutation rates. The fixation time estimated from the two loci models with mutation rates (*μ*) for *A*_2_ and *B*_2_ of either 2.5.10^−7^ (top), 2.5.10^−6^ (middle), or 2.5.10^−5^ (bottom), and the strength of selection on the derived alleles (*s*) is either 0 (left), or 2.10,^−4^ (right). When there is selection, the dominance coefficient (*h*) is either recessive (*h* = 0.1, red), co-dominant (*h* = 0.5, yellow), or dominant (*h* = 0.9, blue). The solid lines in the neutral scenario correspond to analytical approximations: 1/2*μ* (light grey), and Eq (17) (dark grey) (see BDMi Results section for details on the approximations). Each phase portrait shows, for a single simulation, the change in allele frequencies of *A*_2_ and *B*_2_ plotted from the beginning, and then every 100 generations until the fixation of a derived allele. The isoclines represent the expected benefits (warm colours) and costs (cold colours) on population fitness (multiplied by *N*_*e*_, and with a increment of 1). The direction of the arrow indicates the expected allele change (which is the balance between mutation rates and selection), and their size indicates the strength of the change. *N* = 10, 000, *h*_*b*_ = *k*_*b*_ = 0.5, *s*_*b*_ = 10^−3^, *r* = 0.5. 10, 000 iterations.

The 2*μ* term comes from the fact that mutation can occur at both loci. In this case, selfing only shortens the time a mutation needs to spread through the population to fixation (4*N*_*e*_), but it does not affect the waiting time (1/2*μ*). Because the former may be negligible compared to the latter (as implicitly assumed in [43]), selfing barely impacts the fixation time as confirmed by simulations (Fig 6A).

However, when the mutation rate increases (4*N*_*e*_*μ* > 1), the mean time a mutation needs to go to fixation is shorter than 4*N*_*e*_ because mutations of the same type may arise in different individuals in the population, so that multiple mutations can get fixed simultaneously. Taking this effect into account, Kimura [56] showed that, at a single locus, the mean time to fixation under continuous mutation pressure was:

$$\begin{array}{c} T_{Kimura}=\frac{4N_{e}}{4N_{e}\mu -1}\times \left(\gamma +\psi \left(4N_{e}\mu \right)\right) \end{array}$$ (16)

where *γ* is Euler’s constant and *ψ* is the digamma function. Note that (16) converges to (15) for small *N*_*e*_*μ*. Because our BDMi models have two mutation types (for the A and B loci), we cannot use Eq (16) as such because mutations of different types cannot get fixed simultaneously in the populations. As a heuristic argument, we can decompose *T*_*Kimura*_ = *T*_*wait*_ + *T*_*fix*_ as in Eq (3), where *T*_*wait*_ = 1/*μ*. Thus, by using *T*_*fix*_ = *T*_*Kimura*_ − 1/*μ* instead of 4*N*_*e*_ in (15), a more accurate expression under high mutation rate is:

$$\begin{array}{c} T=T_{Kimura}-\frac{1}{2\mu } \end{array}$$ (17)

Importantly, mutations can segregate at the two loci at the same time and be jointly selected against, which is not taken into account in (17). Eq 17 thus serves as a neutral reference to assess whether incompatibilities between mutations segregating within populations affect the fixation time of BDMi mutations, and whether this occurs independently of the population selfing rate.

**Fig 7:**
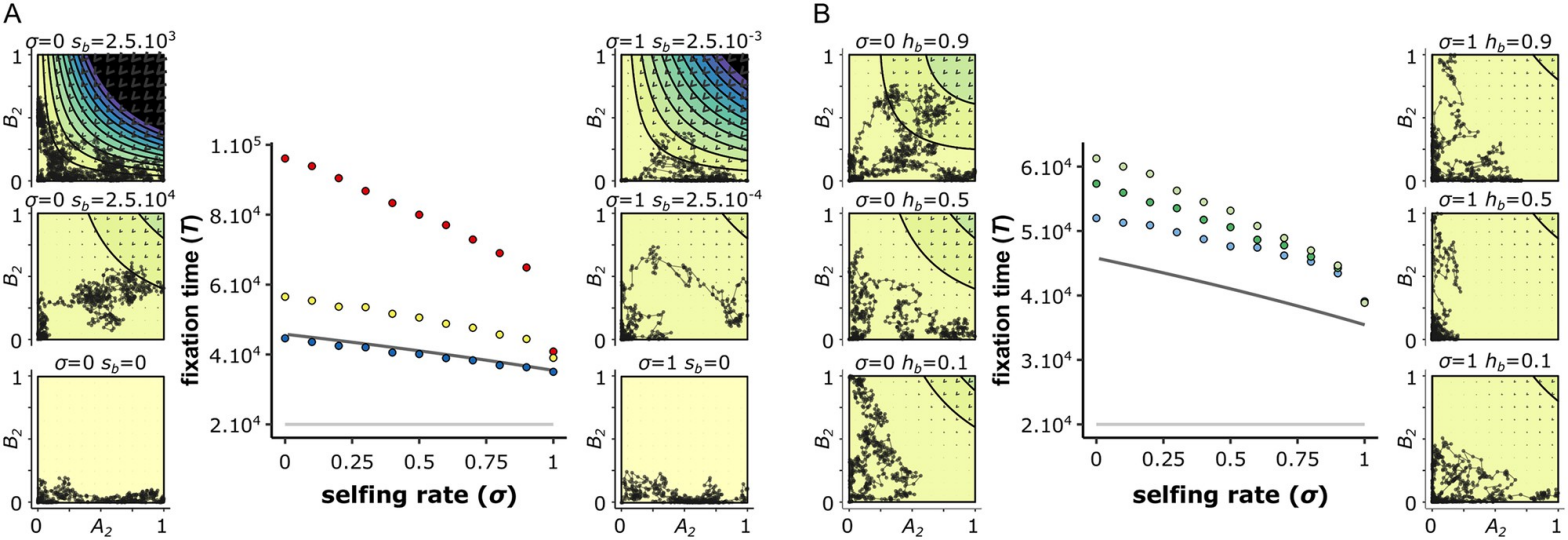
Within-population interferences between BDMi mutations slow down their fixation, and occur less in highly selfing populations (two-locus model). The A panel shows the fixation time of co-dominant BDMi mutations (*h*_*b*_ = 0.5), and with a strength of selection (*s*_*b*_) of either 0 (blue), 2.5.10^−4^ (yellow), or 2.5.10^−3^ (red). The B panel shows the fixation time of BDMi mutations with a strength of selection (*s*_*b*_) of 2.5.10^−4^ that are either recessive (*h*_*b*_ = 0.1; light blue), co-dominant (*h*_*b*_ = 0.5; green), or dominant (*h*_*b*_ = 0.9; light green). The solid lines in the neutral scenario correspond to analytical approximations: 1/2*μ* (light grey), and Eq (17) (dark grey) (see BDMi Results section for details on the approximations). Each phase portrait shows, for a single simulation, the change in allele frequencies of *A*_2_ and *B*_2_ plotted from the beginning, and then every 100 generations until the fixation of a derived allele. The isoclines represent the expected benefits (warm colours) and costs (cold colours) on population fitness (multiplied by *N*_*e*_, and with a increment of 1). The direction of the arrow indicates the expected allele change (which is the balance between mutation rates and selection), and their size indicates the strength of the change. *N* = 10, 000, *k*_*b*_ = *h*_*b*_, *s* = 0, *r* = 0.5. 10, 000 iterations.

Our simulations showed that several BDMi mutations could often segregate together (S9 Fig), and cause incompatibilities within populations (Fig 7A and S7 Fig). We showed that, in outcrossing populations, a high mutation rate and/or a large effective population size slowed down the fixation of BDMi mutations (Fig 5A and 5D). Counter-selection of such segregating incompatibilities hampers the fixation of the derived alleles (Fig 7A), and more dominant incompatibilities (*h*_*b*_ > 0.5; Fig 5B) increase fixation time. Finally, recombination helps forming incompatible *A*_2_*B*_2_ haplotypes and also reduces the accumulation of BDMi mutations (Fig 5C).

Thus, selfing has opposing effects on these dynamics. On the one hand, it increases selection by exposing the incompatible haplotype in homozygous state (especially when *h*_*b*_ is low). On the other hand, it increases drift and reduces genetic shuffling, which reduces the occurrence of the incompatible haplotype. Overall, the second effect dominates and selfing globally reduces the time to fixation of BDMi mutations (Fig 6A, S7 and S9 Figs).

#### Positively selected BDMi mutations (*s* > 0)

When there is positive selection (*s* > 0), the effect of selfing on the accumulation of BDMi mutations depends on the interaction between the mutation rate (*μ*) and the dominance coefficient (*h*) (Fig 6B, and S7 Fig). The results are very similar between local adaptation (positive selection on one locus only) and convergent selection (same positive selection on both loci). We thus only present the case of local adaptation in the main text (see S8 Fig for convergent selection).

When the mutation rate is low, BDMi mutations get fixed as beneficial mutations, whose probability of fixation depends on selfing rate and the dominance coefficient (*h*) [57]. When the mutation is recessive (*i.e*., *h* < 0.5), the probability of fixation increases with selfing. When the mutation is dominant (*i.e*., *h* > 0.5), the probability of fixation decreases with selfing. And, when the mutation is codominant (*i.e*., *h* = 0.5), selfing does not affect the probability of fixation [57]. Therefore, when mutation rate is low, selfing speeds up fixation of recessive BDMi mutations, and slows down fixation of dominant BDMi mutations and can be approximated by equation (13) in [57] (without standing variation, *P*_*sv*_ = 0, their notations) (Fig 6B and S7 Fig). In contrast, when mutation rate increases, selfing speeds up fixation of BDMi mutations regardless of the dominance coefficient (*h*). This is because multiple BDMi mutations segregate in the population (S9 Fig) and cause genetic incompatibilities, hampering more the fixation of BDMi mutations in outcrossing population than in selfing populations, as described above.

#### Effects of background selection on BDMi mutations

**Fig 8:**
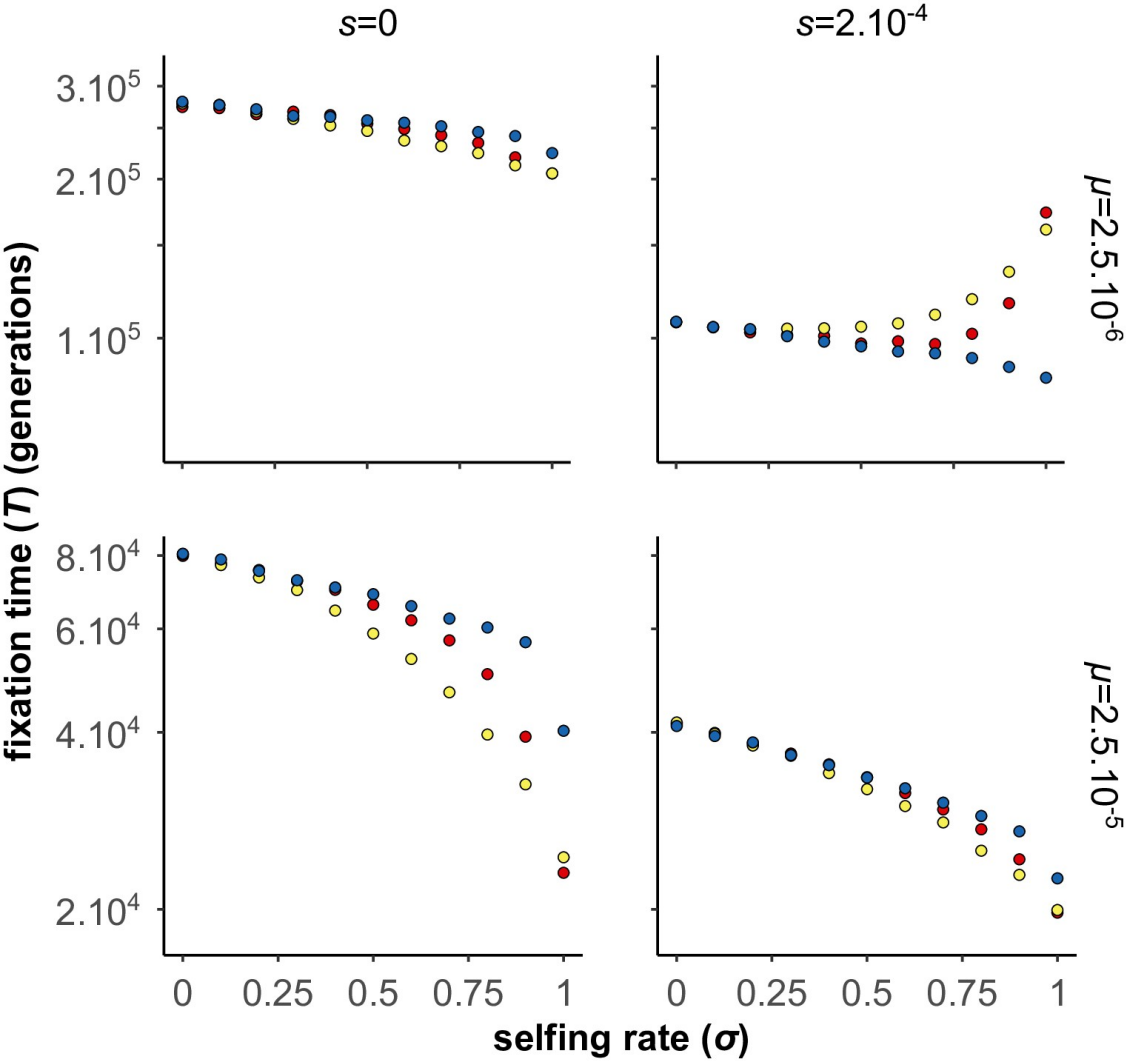
Effects of background selection on the accumulation of BDMi mutations (two-locus model). The graph displays the fixation time, either without (left) or with (right) selection on the derived alleles, and under different scenarios of background selection, ‘linear BG effects’ (yellow) or ‘curved BG effects’ (red), which we compared to a scenario without background selection (blue) (see methods for details on the implementation of background selection effects in our simulations). *N* = 10, 000, *h* = 0.5, *s* = 2.5.10^−4^, *h*_*b*_ = *k*_*b*_ = 0.5, *s*_*b*_ = 10^−3^, *r* = 0.5. 10, 000 iterations.

Background selection has two main effects on the fixation time of BDMi mutations: higher drift makes selection less efficient when selfing increases and the occurrence of segregating mutations at both loci less likely. When there is no selection (*s* = 0), BDMis are less counter selected within populations and accumulate faster under selfing than under outcrossing. Background selection thus reinforces the effect of selfing. When there is selection (*s* > 0), selfing reduces both selection on the beneficial allele and against the incompatible haplotype. When mutation rate is low and selection against the incompatible haplotype is limited, reducing selection on the beneficial allele predominates and selfing slows down the accumulation of BDMi mutations (Fig 8 and S10 Fig). However, for higher mutation rates, reduced selection against the incompatible haplotype predominates and selfing speeds up fixation. Overall, under a wide range of conditions, even with selection, selfing facilitates the accumulation of BDMi mutations.

#### BDMi mutations when selection decreases with selfing rate

**Fig 9:**
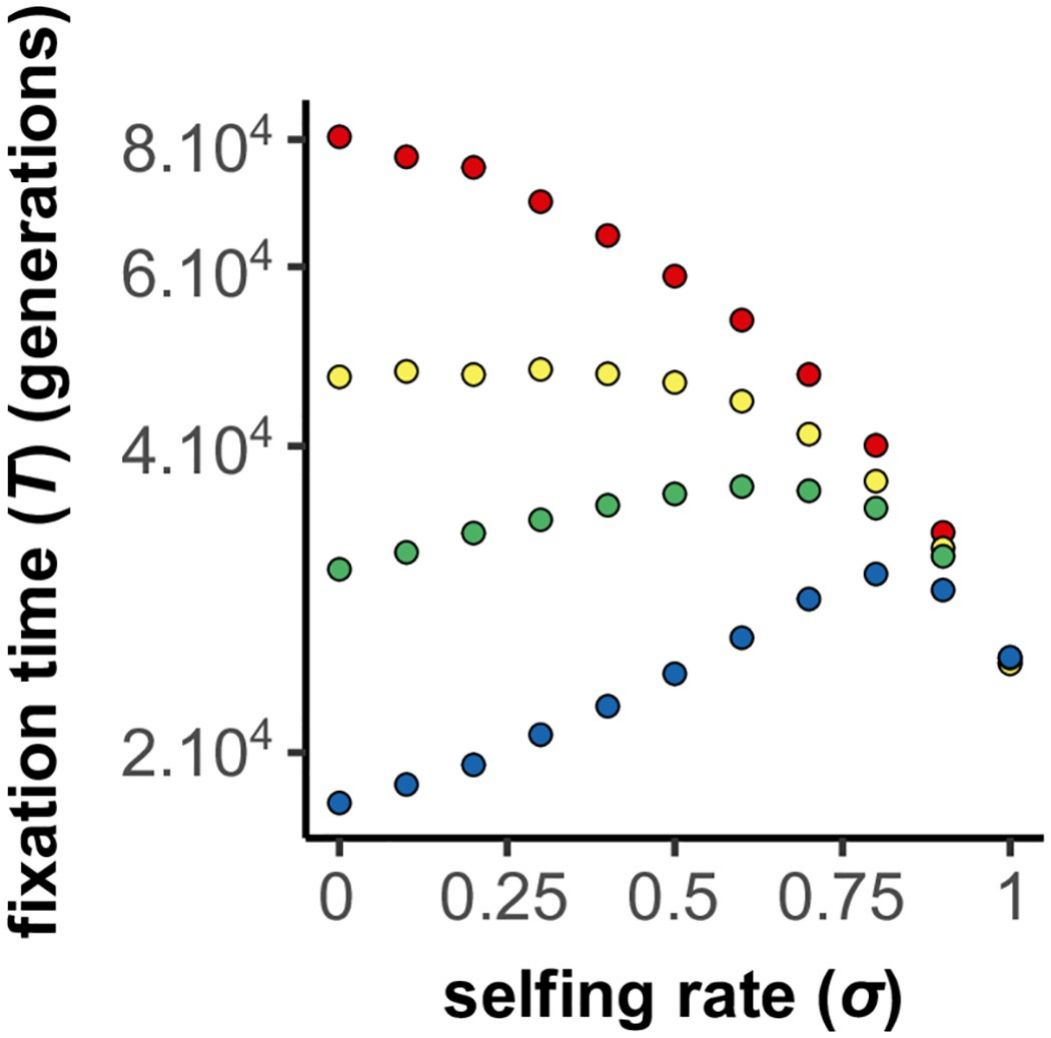
Effects of selfing on the fixation time of BDMi mutations when the strength of selection (*s*) varies with selfing rate (*σ*) (two-locus model). The strength of selection (*s*) decreases with selfing rate as follows: *s* = *s*_0_(1 − *σ*), with *s*_0_ = 0 (red), *s*_0_ = 2.10^−4^ (yellow), *s*_0_ = 4.10^−4^ (green), and *s*_0_ = 8.10^−4^ (blue). *N* = 10, 000, *μ* = 2.5.10^−5^, *h* = 0.5, *h*_*B*_ = *k*_*B*_ = 0.5, *s*_*B*_ = 10^−3^, *r* = 0.5. 10, 000 iterations.

In the previous analyses we considered the selection on the *A*_2_ or *B*_2_ alleles to be independent of selfing (*s* was a constant), as for the adaptation to a new environment. Selfing only modulated the efficacy of selection through its effect on *N*_*e*_ and homozygosity. However, selfing can also directly affect selection, in particular in case of genetic or sexual conflicts [58]. If such conflicts play an important role in the build-up of RI, it has been proposed that selfing may slow down speciation [9]. We did not explore an explicit model involving conflicts, but to mimic such a situation we considered the simple case where selfing directly reduced the selection coefficient: *s* = *s*_0_(1 − *σ*). This is a strong effect as selection vanishes under full selfing. We considered the case of high mutation rate with background selection, which corresponds to the optimal conditions under which selfing promotes speciation. If “conflict” selection is weak, selfing still facilitates the accumulation of BDMi mutations. However, for stronger “conflict” selection (*i.e*., *N*.*s* > 4), despite negative interaction among segregating BDMi, fixation of BDMis is faster under outcrossing than selfing (Fig 9).

## Discussion

The role of mating systems in speciation is an old question, in particular among plant evolutionary biologists [1, 20, 59, 60]. Depending on the underlying mechanisms, selfing has been proposed to either promote or hamper speciation [8, 9]. Surprisingly, despite this long-standing interest, specific models on the role of selfing in the evolution of RI are scarce and mainly concern RI caused by underdominant mutations [44]. Here, we partly filled this gap by expanding previous theoretical work on underdominant mutations, and by considering RI caused by epistatic mutations, such as compensatory mutations and Bateson-Dobzhansky-Muller incompatibility mutations. Overall, we showed that selfing facilitates the accumulation of genetic incompatibilities among populations for a wide range of parameters, and may therefore promote allopatric speciation. Our results further predict that the genomic architecture of reproductive isolation varies with mating system.

### Selfing helps cross fitness valleys

The Bateson-Dobzhansky-Muller model was initially proposed as a possible solution of the puzzling question of the evolution of hybrid incompatibilities as it does not require crossing fitness valleys [61]. However, some mechanisms can facilitate crossing fitness valleys, and it is well known that selfing facilitates the fixation of underdominant mutations [44]. We extended this model to a two-locus fitness landscape (*i.e*., compensatory mutations) for which selfing also helps crossing the valley under most conditions. Selfing has two main effects. First, it increases drift (in particular if background selection is strong). Second, because of reduced recombination and heterozygozity, it limits the breakdown of the new fittest genotype, once it has been created. At the genome scale, assuming several loci for which underdominant fitness landscapes may occur, we showed that the effect of selfing is stronger when there is a highly skewed distribution of the depth of the valleys to be crossed (11). A similar conclusion is also likely for the compensatory mutation model although we did not obtain an equivalent analytical result.

### The role of interference among mutations segregating within populations

In the simplest form of the Bateson-Dobzhansky-Muller model of speciation, genetic incompatibilities occur between derived alleles that are supposed to arise and become fixed independently in different populations [7, 62]. The phase during which BDMi mutations emerge, spread through the population and eventually get fixed is often dismissed (*e.g*., [42]). This necessary phase has recently been argued to have important implications in speciation genetics [63, 64]. Considering that BDMi alleles may segregate in natural populations at polymorphic frequencies allows for instance to better explain (i) why hybrid incompatibility may be variable between different pairs of individuals originating from the same two populations (reviewed in [63]), and (ii) why genetic incompatibilities are widespread within species, as found in *Drosophila melanogaster* [65], *Caenorhabditis elegans* [66], *Arabidopsis thaliana* [67], or the genus *Draba* [18, 19].

To mimic genetic incompatibilities arising from mutations at multiple sites in the genome, we used elevated mutation rates (4*N*_*e*_*μ* > 1) in the two-locus model, which allowed us to dissect the dynamics of multiple incompatible alleles segregating within populations. Under these conditions, we showed that epistatic interactions among segregating BDMis delay their fixation, especially when they are unlinked and not too recessive. These results were validated by multi-locus simulations. This effect was not predicted by previous approximations which showed that, when mutations are rare enough, RI only depends on mutation rate and not on other population parameters and reproductive mode [43]. However, our results are in agreement with some phenomenological models, which assumed that incompatibility was a function of the genetic distance between individuals, and thus can be counter-selected in large polymorphic populations [68].

Our results show that mating system impacts the purging of segregating BDMi mutations, and thereby their fixation time. In selfing populations, the likelihood for two BDMI mutations to arise and be gathered in the same genotype is reduced because of higher genetic drift, reduced polymorphism (including for BDMi mutations), and reduced genetic shuffling. The accumulation of BDMi mutations is thus favoured in selfing populations compared to outcrossing ones—even under scenarios of ecological speciation where the BDMi mutations are positively selected.

### Mating systems and the pace of speciation

Although it has been discussed for decades, whether and how selfing affects the pace of speciation remains unclear. Our results show that selfing overall facilitates the accumulation of underdominant, compensatory, and BDMi mutations in allopatry. Remarkably, this effect may even persist in the face of local adaptation. Overall, selfing broadens the spectrum of genetic incompatibilities that can fix and shortens the time to fixation under many conditions.

However, the pace of accumulation of genetic incompatibilities may not be sufficient to predict the pace of speciation. Indeed, hybrid fitness is indeed not only determined by hybrid incompatibilities, but also by heterosis effects [69–71]. In addition to accelerating the accumulation of hybrid incompatibilities, selfing is also more permissive to the fixation of weakly deleterious mutations degrading the mean population fitness (drift load) [72, 73]. Higher fitness can then be restored by outbreeding between diverged populations, which may promote gene flow, counteracting the effects of incompatibilities and hampering speciation [74]. However, this effect is expected to manifest mainly in the F1 generation [69, 70] and be rather transient [75]. In addition, heterosis generated by the accumulation of deleterious mutations has been shown to have only a weak to moderate effect on hybrid zones maintained by selection [76]. This suggests that selfing should globally facilitate speciation under a wide range of conditions, but this remains to be firmly confirmed by modelling explicitly the balance between hybrid incompatibilities and heterosis over population divergence.

The clear condition under which outcrossing should promote speciation is when speciation is driven by genomic or sexual conflicts, which may vanish under selfing. It is for instance known that sexual conflicts over maternal provisioning during seed development usually are stronger in outcrossers than in selfers (*e.g*., [24]), *i.e*. sexually antagonistic co-evolution of male and female traits is expected to go faster in outcrossing populations, promoting speciation [26]. Our basic model, in which selection decreases linearly with selfing rate, confirms this prediction as soon as antagonistic selection is strong enough (*i.e*., of the order of *N*_*e*_*s* > 5).

Empirical results are so far limited but tend to support our predictions, in particular the accumulation of numerous incompatibilities between recently diverged populations of several selfing Arctic plant species [18, 19], and the phylogeny-based analyses suggesting self-compatible lineages in the Solanaceae have higher speciation rates than self-incompatible [12, 16]. However, the process of speciation in these cases remains poorly known. In Arctic species, divergence in allopatry seems most likely, but in the Solanaceae, selfing may have promoted speciation through limitation of gene flow, which we did not study here.

### Mating systems and the genetic architecture of reproductive isolation

Beyond the effect of mating systems on the pace of speciation, our outcomes clearly suggest that mating system also affects the genetic architecture of speciation. In particular, underdominant and compensatory mutations are expected to be found relatively more often as reproductive barriers among selfers than among outcrossers. Classical examples of genetic modifications leading to underdominant effects include chromosomal rearrangements, which can generate and maintain RI between populations or species [34, 77]. To our knowledge, there are no studies specifically comparing the occurrence of underdominant chromosomal rearrangement in selfing *vs*. outcrossing species. Reproductive isolation due to underdominant chromosomal rearrangements is however more often found in plants than in animals [78], which possibly is due to the higher frequency of selfing among plant species. Our results also suggest that reproductive barriers caused by a few strongly underdominant mutations are more likely to differ between selfers and outcrossers than reproductive barriers caused by many weakly underdominant mutations.

Compensatory effects are often discussed in the context of the evolution of gene expression for which stabilising selection may lead to co-evolution of *cis*- and *trans*-regulatory mutations (*e.g*., a *cis*-regulatory mutation increasing gene expression may be compensated by a *trans*-regulatory mutation decreasing gene expression, or *vice versa*) [79]. Although compensatory mutations are expected to take a long time to become fixed [35, 46], co-evolution of *cis*- and *trans*-regulatory mutations have been found to contribute to RI between outcrossing species of *Drosophila* [36] and mice [38], and also between selfing species of nematode [37].

Finally, our models predict that in outcrossing species, BDMi mutations are more likely to go to fixation when they are clustered (but in repulsion) than when they are widespread. In contrast, we found no specific constraint on genomic location in selfing species such that pairs of incompatible alleles could arise everywhere in a genome. This conclusion is in line with the prediction that genes involved in local adaptation [80] or in domestication [81] should be less clustered under selfing than under outcrossing.

## Conclusions

Our analytical and simulation models show that selfing overall fosters the accumulation of underdominant, compensatory, and BDMi mutations in allopatry. This outcome helps us predict the speciation rates as well as the architecture of RI of selfing *vs*. outcrossing species. Our results provide a theoretical basis for long-standing ideas [1, 5] and are tentatively supported by both phylogenetic studies and crossing experiments—though additional empirical work is needed. Future theoretical work will need to account for the effect of selfing on the stability of RI in the face of gene flow.

## Supporting information

S1 Fig Effects of selfing on the accumulation of underdominant mutations. (PDF)

S2 Fig Effects of selfing on the accumulation of a pair of compensatory mutations when the strength of the deleterious effect (*s*_*c*_) is low (two-locus model). (PDF)

S3 Fig Effects of selfing on the accumulation of a pair of compensatory mutations (two-locus model). (PDF)

S4 Fig Effects of selfing rate on the fitness of a population, and the path taken on the fitness landscape, over the 4200 generations preceding the fixation of the pair of compensatory mutation (two-locus model). (PDF)

S5 Fig Effects of background selection on the accumulation of compensatory mutations (two-locus model). (PDF)

S6 Fig Effects of selfing on the accumulation of compensatory mutations (multi-locus model). (PDF)

S7 Fig Effects of selfing and selection on the accumulation of BDMi mutations (multi-locus model). (PDF)

S8 Fig Effects of selfing and convergent selection on the fixation time of BDMi mutations (two-locus model). (PDF)

S9 Fig Effects of selfing and selection on the fixation time and the number of BDMi mutations segregating in a population (multi-locus model). (PDF)

S10 Fig Effects of selfing, selection and background selection on the time to fixation fixation of BDMi mutations (multi-locus model). (PDF)

S1 Appendix Mathematical derivations. (PDF)

S1 Scripts Mathematica notebook, C++ scripts, SLiM scripts. (ZIP)
