# Supplementary material for: Mating system and speciation I: Accumulation of genetic incompatibilities in allopatry
Source: PLoS Genet. 2022 Dec 15;18(12):e1010353. doi: 10.1371/journal.pgen.1010353 (PMC9799327; doi:10.1371/journal.pgen.1010353)
Supplement: S1 Fig — (PDF) [file pgen.1010353.s001.pdf]

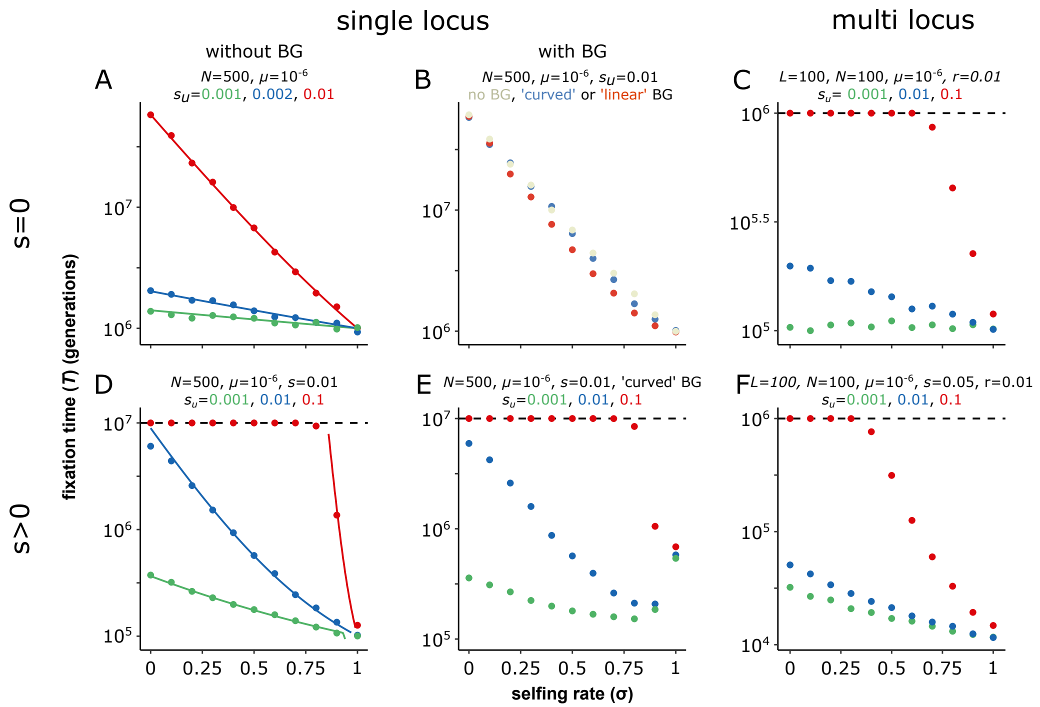

Fig S1. Effects of selfing on the accumulation of underdominant mutations. Outcomes of the simulations (circles) for the number of generations to fixate either one derived allele (single locus; A, B, D, E), or ten derived alleles (multi-locus models; C, F). The top row corresponds to symmetrical underdominant mutations (*i.e.*, $s = 0$). The bottom row corresponds to asymetrical underdominant mutations (*i.e.*, $s > 0$). Single locus simulations were performed either with (A, D) or without (B, E) background selection, BG. The dashed horizontal lines correspond to the threshold after which simulations stopped to avoid computing burden. $1,000$ iterations. Lines on A and D correspond to numerical approximations.
