# Supplementary material for: Mating system and speciation I: Accumulation of genetic incompatibilities in allopatry
Source: PLoS Genet. 2022 Dec 15;18(12):e1010353. doi: 10.1371/journal.pgen.1010353 (PMC9799327; doi:10.1371/journal.pgen.1010353)
Supplement: S2 Fig — (PDF) [file pgen.1010353.s002.pdf]

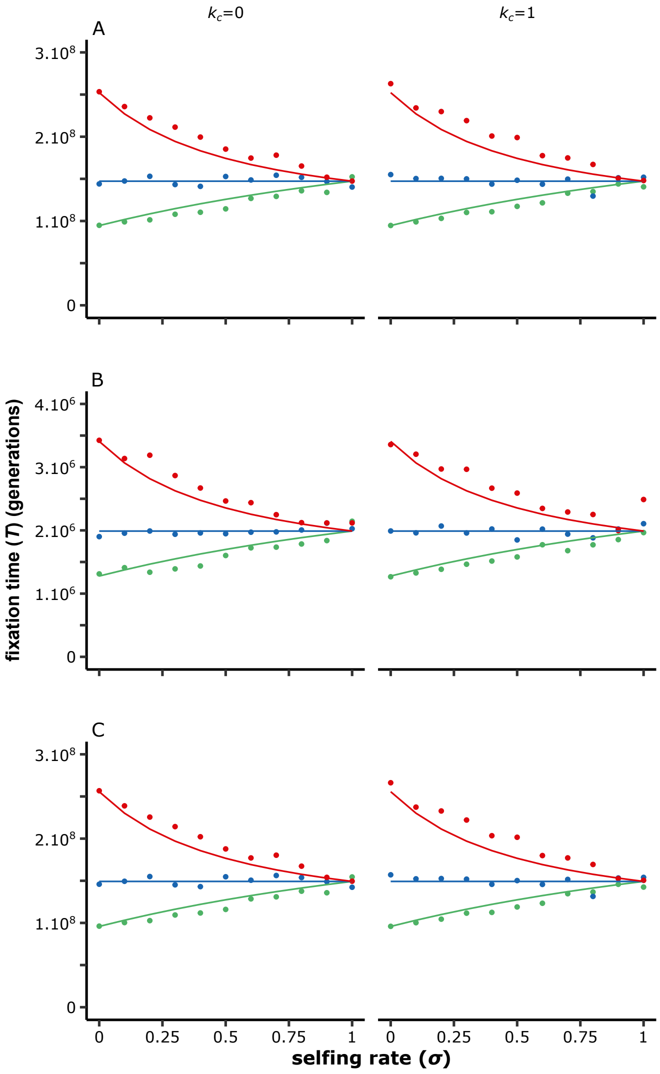

Fig S2. Effects of selfing on the accumulation of a pair of compensatory mutations when the strength of the deleterious effect (s_c) is low (two-locus model). The fixation time of the first (deleterious) mutation (A), of the second (compensatory) mutation once the first mutation is fixed (B), and of the pair of mutations (C) (mind the different y-scales). The dots are the simulation outcomes. The lines are the numerical predictions (see Mathematica file). The color stands for the coefficient of dominance of the deleterious effect, which is either recessive ($h_c = 0.1$, green), codominant ($h_c = 0.5$, blue) or dominant ($h_c = 0.9$, red). The coefficient of dominance of the double heterozygotes (k_c) is set to 0 (left) or 1 (right). Selfing rate (σ) ranges from 0 to 1, with a 0.1 increment. $N = 1,000$, $\mu = 10^{-7}$, $s_c = 0.0025$, $r = 0.5$. 1,000 iterations.
