# Supplementary material for: Mating system and speciation I: Accumulation of genetic incompatibilities in allopatry
Source: PLoS Genet. 2022 Dec 15;18(12):e1010353. doi: 10.1371/journal.pgen.1010353 (PMC9799327; doi:10.1371/journal.pgen.1010353)
Supplement: S3 Fig — (PDF) [file pgen.1010353.s003.pdf]

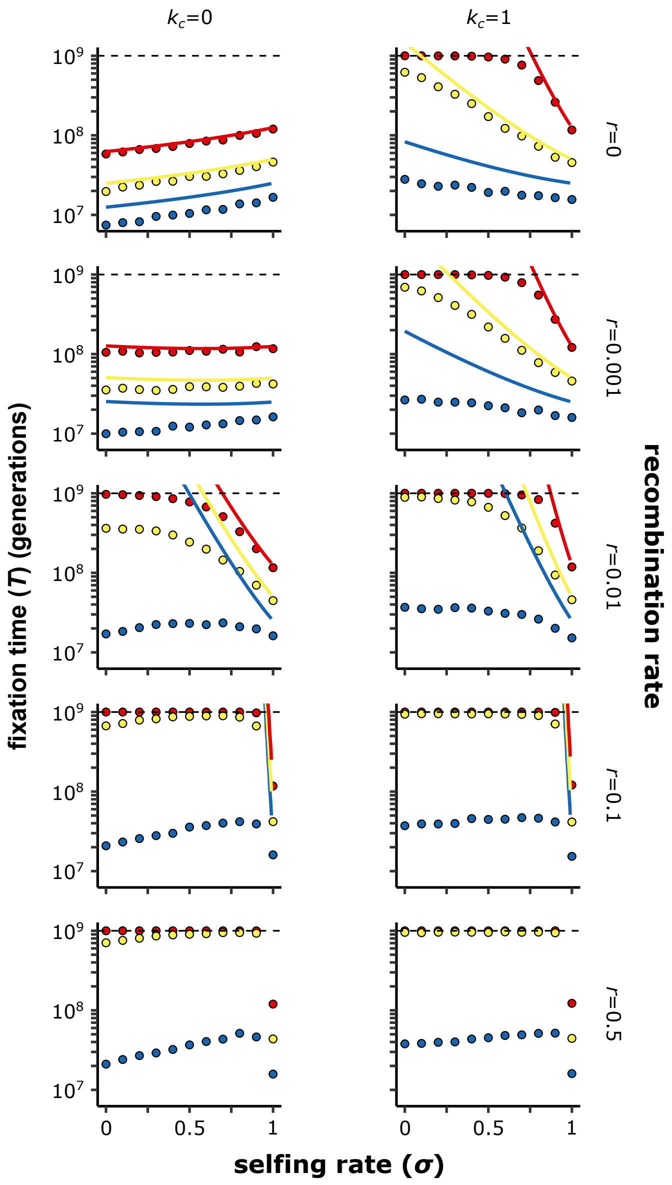

Fig S3. Effects of selfing on the accumulation of a pair of compensatory mutations (two-locus model). Analytical approximations (lines; eq. 14) and outcomes of the two-locus simulations (dots) for the mean number of generations needed to fixate a pair of compensatory mutations. The recombination rate between the two-locus (r) ranges from 0 (top) to 0.5 (down), and the coefficient of dominance of the double heterozygotes (k_c) is set to 0 (left) or 1 (right). The strength of the deleterious effect (s_c) is set to 0.005 (blue), 0.010 (yellow), or 0.025 (red). The threshold after which simulations terminate was set to $10^9 generations$ (dashed line). Selfing rate (σ) ranges from 0 to 1, with a 0.1 increment. $N = 1,000$, $\mu = 10^{-5}$, $h_c = 0.5$. 1000 iterations.
