# Supplementary material for: Mating system and speciation I: Accumulation of genetic incompatibilities in allopatry
Source: PLoS Genet. 2022 Dec 15;18(12):e1010353. doi: 10.1371/journal.pgen.1010353 (PMC9799327; doi:10.1371/journal.pgen.1010353)
Supplement: S4 Fig — (PDF) [file pgen.1010353.s004.pdf]

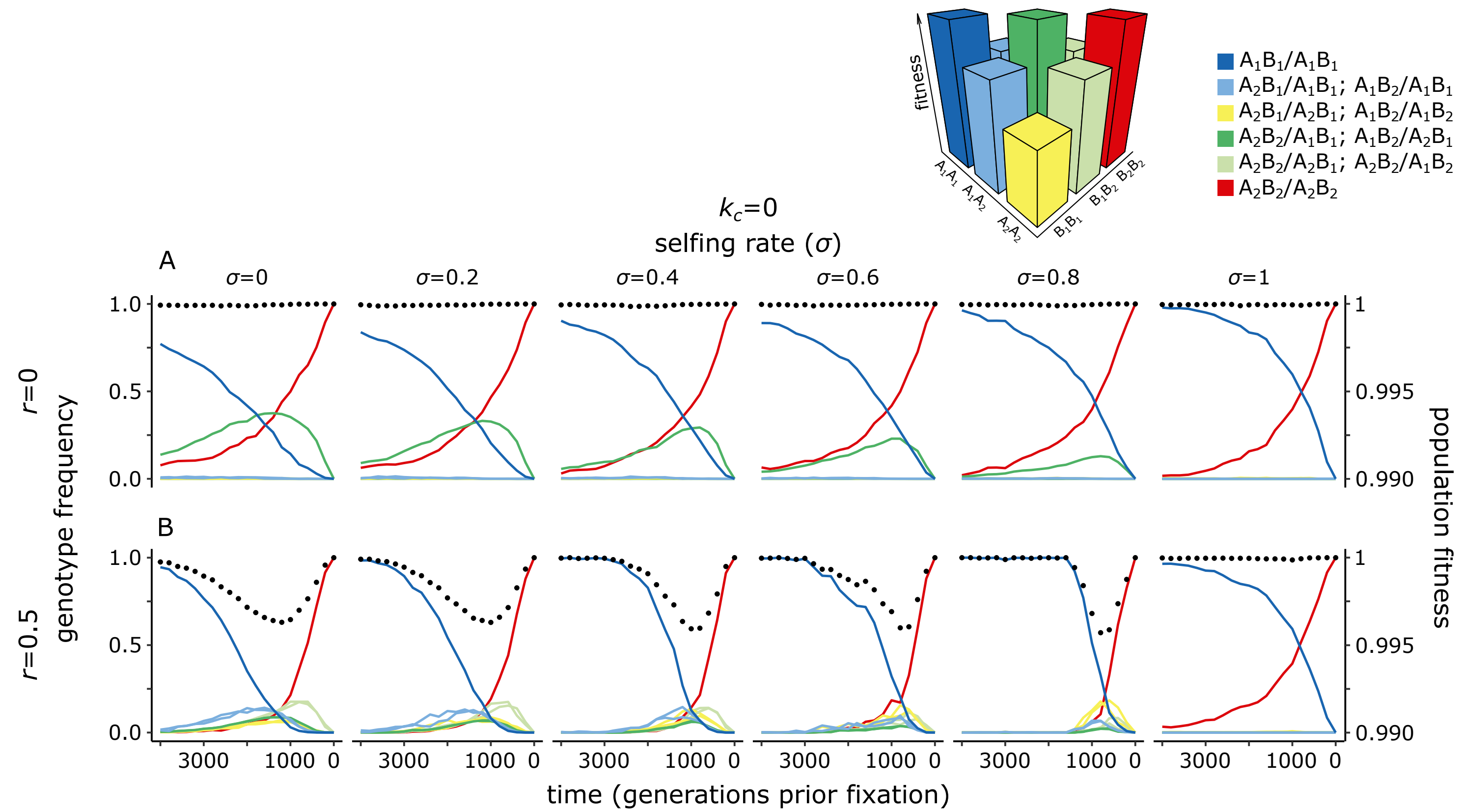
fitness
A_1A_1 A_1A_2 A_2A_2
B_1B_1 B_1B_2 B_2B_2
A_1B_1/A_1B_1
A_2B_1/A_1B_1; A_1B_2/A_1B_1
A_2B_1/A_2B_1; A_1B_2/A_1B_2
A_2B_2/A_1B_1; A_1B_2/A_2B_1
A_2B_2/A_2B_1; A_2B_2/A_1B_2
A_2B_2/A_2B_2
k_c=0
selfing rate (σ)
A
σ=0 σ=0.2 σ=0.4 σ=0.6 σ=0.8 σ=1
r=0
r=0.5
genotype frequency
population fitness
B
1.0 0.5 0.0
1 0.995 0.990
3000 1000 0
time (generations prior fixation)

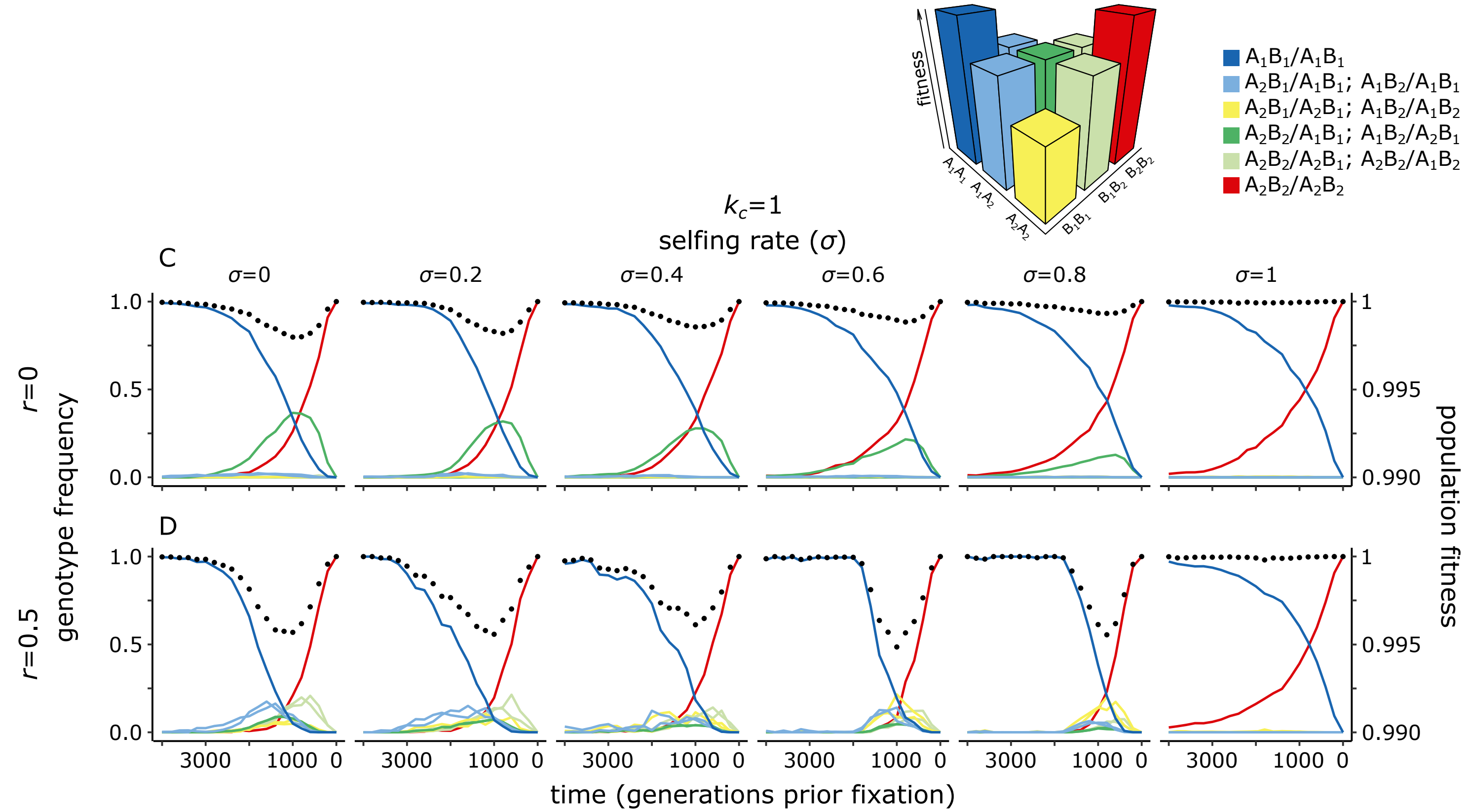

Fig S4. Effects of selfing rate on the fitness of a population, and the path taken on the fitness landscape, over the 4200 generations preceding the fixation of the pair of compensatory mutation (two-locus model). Outcomes of the two-locus simulations showing population fitness (black dots, right y axis) and the frequencies of the 10 possible genotypes on the two-locus fitness landscapes (solid lines, left y axis). Selfing rate (σ) ranges from 0 (left) to 1 (right), with a 0.2 increment. The coefficient of dominance of the double heterozygotes (k_c) is set to 0 (A, B) or 1 (C, D), and the recombination rate between the two loci is set to 0 (A, C) or to 0.5 (B, D). $N = 1,000$, $\mu = 10^{-5}$, $h_c = 0.5$, $s_c = 0.01$. 100 iterations.
