# Supplementary material for: Mating system and speciation I: Accumulation of genetic incompatibilities in allopatry
Source: PLoS Genet. 2022 Dec 15;18(12):e1010353. doi: 10.1371/journal.pgen.1010353 (PMC9799327; doi:10.1371/journal.pgen.1010353)
Supplement: S5 Fig — (PDF) [file pgen.1010353.s005.pdf]

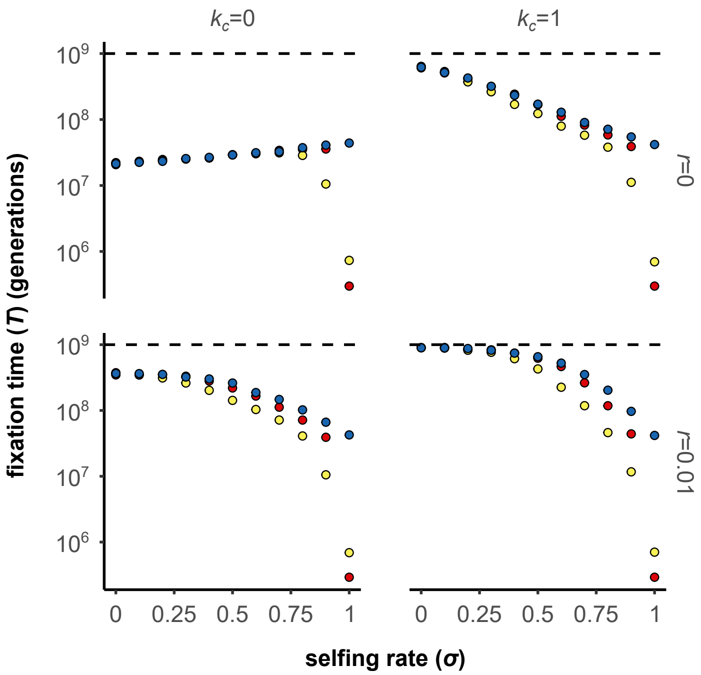

Fig S5. Effects of background selection on the accumulation of compensatory mutations (two-locus model). The graph displays the fixation time estimated from the two-locus models with a coefficient of dominance of the double heterozygotes (k_c) set to 0 (left) or 1 (right), and under different scenarios of background selection, 'linear BG effects' (yellow) or 'curved BG effects' (red), which we compared to a scenario without background selection scenario (blue) (see methods for details on the implementation of background selection effects in our simulations). $N = 1,000$, $\mu = 10^{-5}$, $h_c = 0.5$, $s_c = 10^{-2}$, $r = 0.5$. $10,000$ iterations
