# Supplementary material for: Mating system and speciation I: Accumulation of genetic incompatibilities in allopatry
Source: PLoS Genet. 2022 Dec 15;18(12):e1010353. doi: 10.1371/journal.pgen.1010353 (PMC9799327; doi:10.1371/journal.pgen.1010353)
Supplement: S6 Fig — (PDF) [file pgen.1010353.s006.pdf]

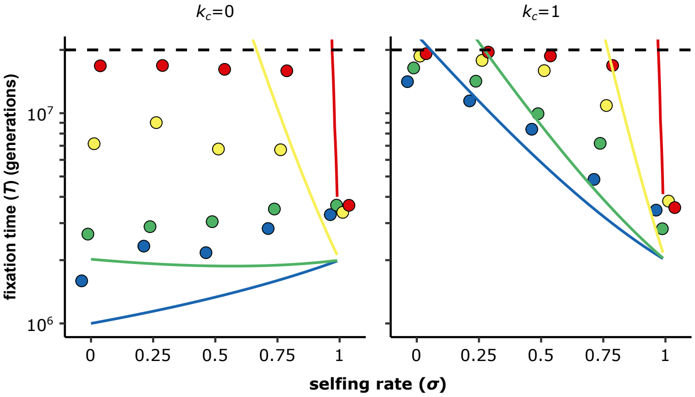

Fig S6. Effects of selfing on the accumulation of compensatory mutations (multi-locus model). The graph shows the two-locus analytical approximations (lines; eq. 14) and the outcomes of the multi-locus simulations (dots) for the mean number of generation needed for the fixation of at least two compensatory mutations. The threshold after which the simulation terminates is set to 2.10^7 generations (dashed lines). The coefficient of dominance of the double heterozygotes (k_c) is set to 0 (left) or 1 (right), and the recombination rate between each locus is set to 0 (blue), 0.0001 (green), 0.001 (yellow), or 0.01 (red). Selfing rate (σ) ranges from 0 to 1, with a 0.25 increment. $L = 100$, $N = 200$, $\mu = 10^{-6}$, $h_c = 0.5$, $s_c = 0.04$. 100 iterations.
