# Supplementary material for: Mating system and speciation I: Accumulation of genetic incompatibilities in allopatry
Source: PLoS Genet. 2022 Dec 15;18(12):e1010353. doi: 10.1371/journal.pgen.1010353 (PMC9799327; doi:10.1371/journal.pgen.1010353)
Supplement: S7 Fig — (PDF) [file pgen.1010353.s007.pdf]

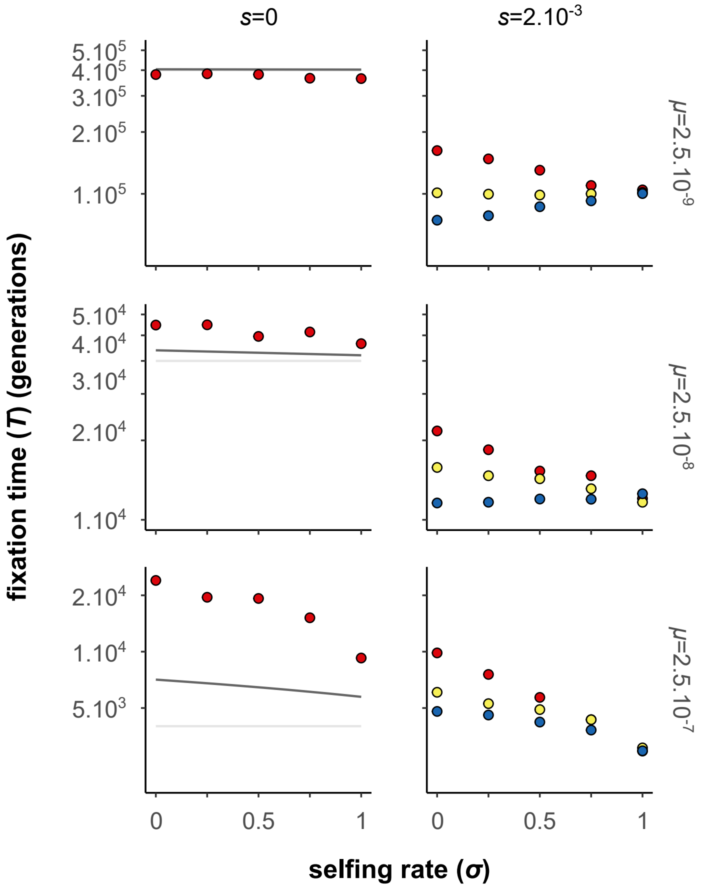

Fig S7. Effects of selfing and selection on the accumulation of BDMi mutations (multi-locus model). The panel displays the fixation time estimated when the mutation rate, μ, is either $2.5.10^{-9}$ (top), $2.5.10^{-8}$ (middle), or $2.5.10^{-7}$ (bottom). The strength of selection on the derived alleles, s, is either 0 (left), or 2.10^{-3} (right). When there is selection, the dominance coefficient of the derived alleles, h, is either recessive (0.1, red), codominant (0.5, yellow), or dominant (0.9, blue). The solid lines in the neutral scenario correspond to analytical approximations: $1/2\mu$ (light grey), and equation (17) (dark grey) (see BDMi Results section for details on the approximations). $L = 1,000$, $N = 1,000$, $h_b = k_b = 0.5$, $s_b = 10^{-2}$, $r = 10^{-3}$. 1,000 iterations.
