# Supplementary material for: Mating system and speciation I: Accumulation of genetic incompatibilities in allopatry
Source: PLoS Genet. 2022 Dec 15;18(12):e1010353. doi: 10.1371/journal.pgen.1010353 (PMC9799327; doi:10.1371/journal.pgen.1010353)
Supplement: S8 Fig — (PDF) [file pgen.1010353.s008.pdf]

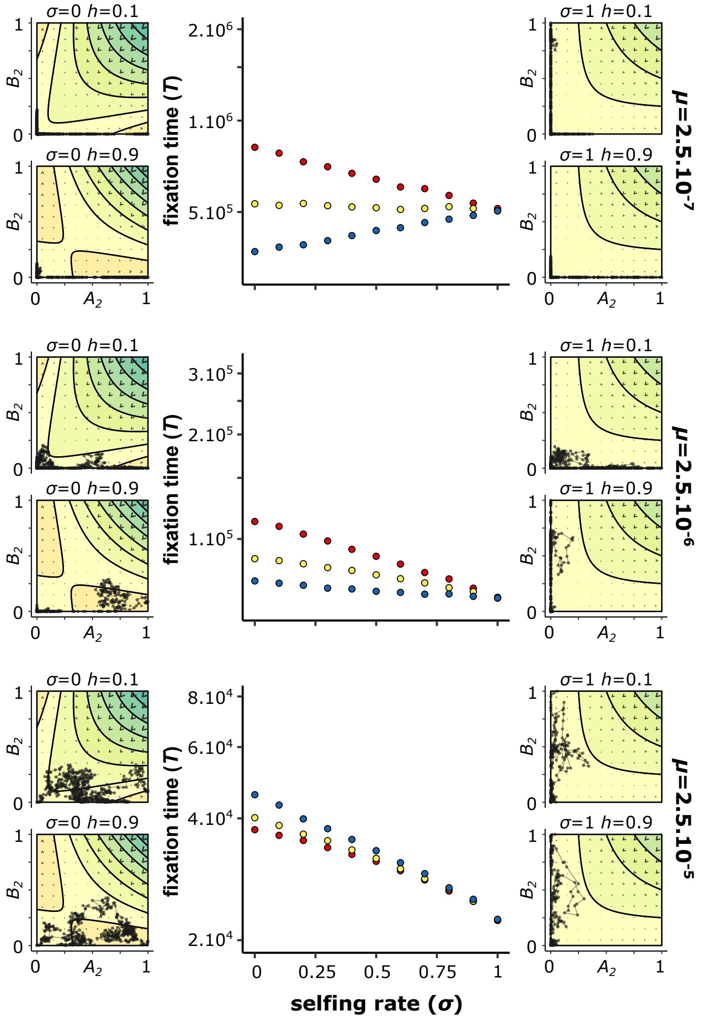

Fig S8. Effects of selfing and convergent selection on the fixation time of BDMi mutations (two-locus model).. The fixation time estimated from the two loci models with mutation rates (μ) for A_2 and B_2 of either $2.5.10^{-7}$ (top), $2.5.10^{-6}$ (middle), or $2.5.10^{-5}$ (bottom), and both loci are under positive selection ($s_A = s_B = 2.10,^{-4}$). The dominance coefficient (h) is either recessive ($h = 0.1$, red), co-dominant ($h = 0.5$, yellow), or dominant ($h = 0.9$, blue). Each phase portrait shows, for a single simulation, the change in allele frequencies of A_2 and B_2 plotted from the beginning, and then every 100 generations until the fixation of a derived allele. The isoclines represent the expected benefits (warm colours) and costs (cold colours) on population fitness (multiplied by N_e, and with a increment of 1). The direction of the arrow indicate the expected allele change (which is the balance between mutation rates and selection), and their size indicates the strength of the change. $N = 10,000$, $h_b = k_b = 0.5$, $s_b = 10^{-3}$, $r = 0.5$. $10,000$ iterations.
