# Supplementary material for: Mating system and speciation I: Accumulation of genetic incompatibilities in allopatry
Source: PLoS Genet. 2022 Dec 15;18(12):e1010353. doi: 10.1371/journal.pgen.1010353 (PMC9799327; doi:10.1371/journal.pgen.1010353)
Supplement: S9 Fig — (PDF) [file pgen.1010353.s009.pdf]

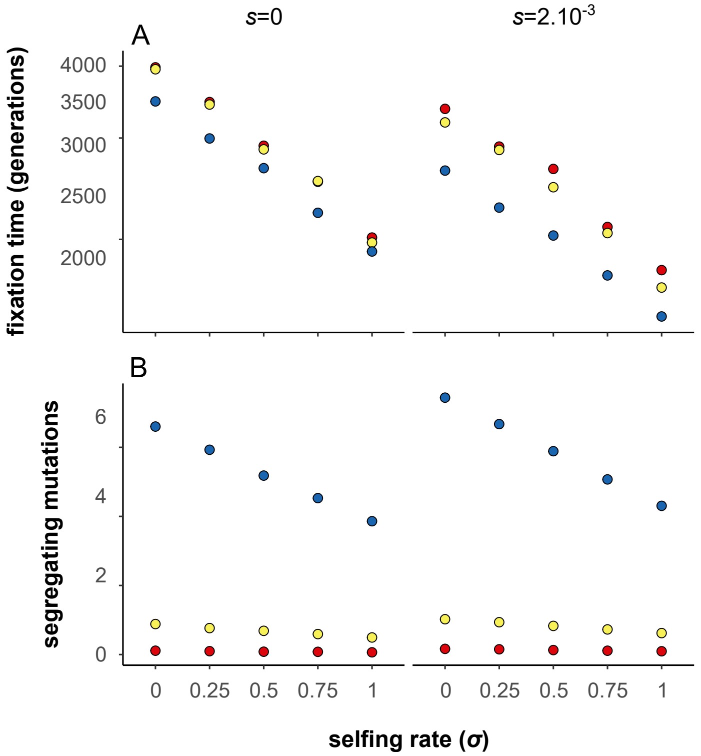

Fig S9. Effects of selfing and selection on the fixation time and the number of BDMi mutations segregating in a population (multi-locus model). The panel displays (A) the time a mutation takes to fixate (fixation time) and (B) the mean number of BDMi mutations segregating in a population sampled every 100 generations. The mutation rate, μ, is either $2.5.10^{-9}$ (red), $2.5.10^{-8}$ (yellow), or $2.5.10^{-7}$ (blue). The strength of selection on the derived alleles, s, is either 0 (left), or 2.10^{-3} (right). $L = 1,000$, $N = 1,000$, $h = 0.5$, $h_b = k_b = 0.5$, $s_b = 10^{-2}$, $r = 10^{-3}$. $1,000$ iterations.
