# Supplementary material for: Mating system and speciation I: Accumulation of genetic incompatibilities in allopatry
Source: PLoS Genet. 2022 Dec 15;18(12):e1010353. doi: 10.1371/journal.pgen.1010353 (PMC9799327; doi:10.1371/journal.pgen.1010353)
Supplement: S10 Fig — (PDF) [file pgen.1010353.s010.pdf]

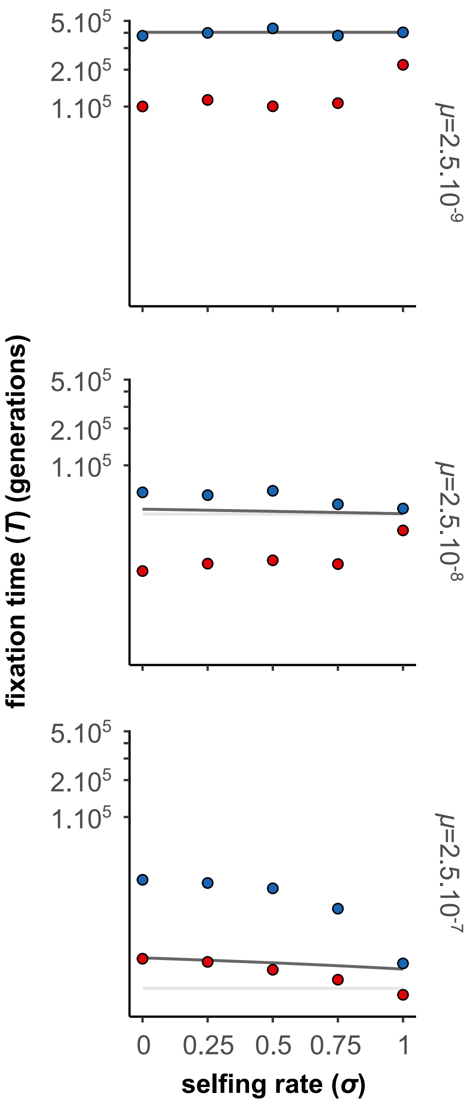

Fig S10. Effects of selfing, selection and background selection on the time to fixation fixation of BDMi mutations (multi-locus model). The graph displays the mean fixation time of BDMi mutations when the strength of selection on the derived alleles, s, is either equal to 0 (blue) or to 2.10^{-3} b(red). The mutation rate, μ, is either $2.5.10^{-9}$ (top), $2.5.10^{-8}$ (middle), or $2.5.10^{-7}$ (bottom). In addition to the BDMi mutations, deleterious mutations with a coefficient of dominance of 0.1 and a strength of selection of -10^{-3} occured at a $5.10^{-}3$ rate. The solid lines in the neutral scenario correspond to analytical approximations: $1/2\mu$ (light grey), and equation (17) (dark grey) (see BDMi Results section for details on the approximations). $L = 1,000$, $N = 1,000$, $h = 0.5$, $h_b = k_b = 0.5$, $s_b = 10^{-2}$, $r = 10^{-3}$. 100 iterations.
