# Supplementary material for: Mating system and speciation I: Accumulation of genetic incompatibilities in allopatry
Source: PLoS Genet. 2022 Dec 15;18(12):e1010353. doi: 10.1371/journal.pgen.1010353 (PMC9799327; doi:10.1371/journal.pgen.1010353)
Supplement: S1 Appendix — (PDF) [file pgen.1010353.s011.pdf]

Mating system and speciation I: the accumulation of genetic incompatibilities in allopatry

# - Supplementary Material -

The corresponding Mathematica notebook is also provided

Lucas Marie-Orleach, Christian Brochmann and Sylvain Glémin

6th December 2022

A1 Underdominance

A1.1 Model

We consider a population of size N reproducing with partial selfing in proportion σ. The effective size of the population is [1, 2]:

$$N_e = \frac{N}{1+F} \tag{A1}$$

where F is the Wright's fixation index which neutral expectation is:

$$F = \frac{\sigma}{2-\sigma} \tag{A2}$$

We consider a single bi-allelic locus, with the ancestral allele A_1 that can mutate in the derived allele A_2 at rate μ. We note the fitness of genotypes A_1A_1, A_1A_2, and A_2A_2, 1, $1-s_u$, and $1+s$, respectively, and x the frequency of allele A_2. The change in allelic frequencies in one generation is given by:

$$\begin{aligned}\Delta x &= x(1-x)\Big((1-F)(sx - s_u(1-2x) + Fs\Big)/\bar{W} \\ &\approx x(1-x)\Big((1-F)(sx - s_u(1-2x) + Fs\Big) \end{aligned} \tag{A3}$$

where $\bar{W}$ is the mean fitness of the population. Under weak selection $\bar{W} \approx 1$ (second line of A3) and F can be equated to its neutral expectation (A2). Equation

(A3) can also be written as:

$$\Delta x = (1-F)Sx(1-x)(x-x_{eq}) \quad \text{if } F<1$$
$$= sx(1-x) \quad \text{if } F=1 \tag{A4}$$

where $S = s + 2s_u$, representing the total amount of selection (s_u between A_1A_1 and A_1A_2 and $s_u + s$ between A_1A_2 and A_2A_2), and x_{eq} is the internal (unstable) equilibrium:

$$x_{eq} = \frac{(1-F)s_u - Fs}{(1-F)(2s_u+s)} \tag{A5}$$

This internal equilibrium exist ($0 \leq x_{eq} \leq 1$) if $F \leq s/(s+s_u)$. Above this threshold, selection becomes directional and positive, and for F exactly equal to this threshold, equation (A3) becomes:

$$\Delta x = \frac{s}{s+s_u}(s+2s_u)x^2(1-x) \tag{A6}$$

which is equivalent to selection on a fully recessive allele with selective advantage $s(s+2s_u)/(s+s_u)$.

A1.2 Probability and time to fixation

Noting $M_{\delta x} = \Delta x$ the expected infinitesimal change in allelic frequency and $V_{\delta x} = \frac{x(1-x)}{2N_e}$ is the infinitesimal variance we define the so-called Green function as:

$$G(x) = e^{-\int 2M_{\delta x}/V_{\delta x}dx} \tag{A7}$$

The probability of fixation of a single A_2 mutant is then given by Kimura 1962 [3]:

$$P_{fix} = \frac{\displaystyle\int_0^{1/2N} G(x)dx}{\displaystyle\int_0^1 G(x)dx} \tag{A8}$$

This solves to:

$$P_{fix} = \frac{\operatorname{erf}\left(x_{eq}\sqrt{2N_eS(1-F)}\right) - \operatorname{erf}\left(\left(x_{eq} - \frac{1}{2N}\right)\sqrt{2N_eS(1-F)}\right)}{\operatorname{erf}\left((1-x_{eq})\sqrt{2N_eS(1-F)}\right) + \operatorname{erf}\left(x_{eq}\sqrt{2N_eS(1-F)}\right)} \tag{A9}$$

where erf is the error function. Under recurrent mutations, fixation is certain and we are interested in the time to ultimate fixation. It can be obtained using Kimura 1980 [4]. The infinitesimal mean change is now given by $M_{\delta x} = \Delta x + \mu(1-x)$ where the additional term corresponds to recurrent mutation. We then plug $M_{\delta x}$ into the Green function (A7) and the time to ultimate fixation under recurrent mutation is given by:

$$T_{fix} = \int_0^1 \int_0^x 4N_e \frac{G(z)}{(1-z)z} dz \frac{1}{G(x)} dx \tag{A10}$$

No close form solution exist for (A10) so numerical integration must be carried out (see *Mathematica* notebook). However, we can obtain an approximation as follows (see also [5]). The time to ultimate fixation can be decomposed into two parts: the waiting time for the appearance of the mutation destined to be fixed plus the time to fixation conditioned on the fact that fixation will occur. Because of underdominance the waiting time is expected to be much longer than the conditioned fixation time, which can be neglected. The time to ultimate fixation can thus be

approximated by:

$$T_{fix} \approx \frac{1}{2NuP_{fix}} \tag{A11}$$

For most parameters, (A11) is very accurate.

A1.3 Thresholds for near-neutrality

Assuming $x << 1$ in equation (A3) shows that a rare underdominant mutation behaves almost like a deleterious allele with a deleterious heterozygote effect $(1-F)Sx_{eq}$ if $x_{eq} > 0$. For $x_{eq} < 0$ the mutation is positively selected for and can easily fix, which corresponds to the threshold $F \geq s_u/(s+s_u)$ (see above). Alternatively, for a given selfing rate, mutations with an heterozygote effects lower than the following threshold can easily fix:

$$s_u^{lim} = s\frac{F}{1-F} \tag{A12}$$

This threshold vanishes to 0 when $s = 0$ and all mutants initially behave as deleterious.

When $s = 0$ we can consider a less stringent threshold as follows. When a mutant arises, $x << 0$ so:

$$\Delta x \approx -(1-F)s_u x \tag{A13}$$

This is equivalent to negative genic selection with an effective selection coefficient $s_e = (1-F)s_u$. We can consider that the mutation behaves almost neutraly when $2N_e s_e \leq 1$, which can be expressed as: $2N(1-\sigma)s_u \leq 1$. So the nearly neutral

threshold is simply given by:

$$s_u^{nn} = \frac{1}{2N(1-\sigma)} \tag{A14}$$

A1.4 Distribution of deleterious effects

We now consider that the scaled heterozygote effects, $2Ns_u$ are not fixed but follow a gamma distribution with mean $\gamma = 2N\overline{s}_u$ and shape β, with *pdf* given by:

$$\phi(z) = \frac{\left(\frac{\gamma}{\beta}\right)^{-\beta} z^{\beta-1} e^{-\frac{\beta z}{\gamma}}}{\Gamma(\beta)} \tag{A15}$$

The proportion of symmetrical underdominant mutations ($s = 0$) that can fix (with a reasonable chance), ρ, is thus given by:

$$\begin{aligned} p(\sigma) &= \int_0^{1/2N(1-\sigma)} \phi(z)dz \\ &= 1 - \frac{\Gamma\left(\beta, \frac{\beta}{\gamma-\gamma\sigma}\right)}{\Gamma(\beta)} \end{aligned} \tag{A16}$$

Assuming that $\beta << \gamma$ and taking Taylor expansion of (A16) in β/ρ close to 0 we get:

$$p(\sigma) \approx \frac{\left(\frac{\beta}{\gamma(1-\sigma)}\right)^{\beta}}{\Gamma(\beta+1)} \tag{A17}$$

The ratio $\rho(\sigma) = p(\sigma)/p(0)$ gives the relative excess of the proportion of mutants that can fix compared to an outcrossing population:

$$\rho(\sigma) = \frac{\Gamma(\beta) - \Gamma\left(\beta, \frac{\beta}{\gamma-\gamma\sigma}\right)}{\Gamma(\beta) - \Gamma\left(\beta, \frac{\beta}{\gamma}\right)} \tag{A18}$$

Using the same approximation that $\beta << \gamma$ we obtain the very simple formula:

$$\rho(\sigma) \approx (1-\sigma)^{-\beta} \tag{A19}$$

Numerical and simulations results show that these two expressions also very accurately approximate the excess of probability of fixation (or reduction in fixation time) compared to an outcrossing population: $\frac{\int_0^\infty P_{fix}\phi(S)dS}{\int_0^\infty P_{fix}\phi(S)dS\Big|_{\sigma=0}}$ (or the inverse for fixation time).

A2 Compensatory mutations

A2.1 Model

We now consider a model of compensatory mutations at two loci with two alleles, where two haplotypes are equally fit, A_1B_1 (haplotype 1) and A_2B_2 (haplotype 4), but the intermediate paths, A_1B_2 (haplotype 2) and A_2B_1 (haplotype 3) are deleterious. Alike in the single underdominant model described above, the evolution of pairs of compensatory mutations requires to cross a fitness valley. For simplicity we consider a symmetrical model and we set the fitness of the genotypes as follows:

$$\begin{aligned}
&w_{11} = w_{44} = 1 \\
&w_{22} = w_{33} = 1-s \\
&w_{12} = w_{13} = w_{24} = w_{34} = 1-hs \\
&w_{14} = w_{23} = 1-hks
\end{aligned} \tag{A20}$$

where the subscript ij denotes the genotype formed with haplotypes i and j, and $s \geq 0$ and $0 \leq h \leq 1$ are respectively the strength and the coefficient of dominance of the deleterious effects of each mutation, and k is the coefficient of dominance for the double heterozygote genotype $A_1A_2B_1B_2$. In the main text we added the subscript c to these coefficient to distinguish with the coefficients in the BDMi model. Here we remove them to ease the reading.

We build upon previous haploid models [6, 7] and extend it to diploidy with partial selfing. Previous works have shown that little recombination strongly prevent the fixation of compensatory mutations by breaking down double mutants. Under the assumption of weak recombination, we can only follows the four haplotypes and assume that genotype frequencies are obtained using multi-allelic single locus theory. We note X_i the frequency of haplotype i, and G_{ij} the frequency of genotype ij. This makes the system more tractable than the general system of ten equations presented in the main text. After meiosis, haplotype frequencies are given by:

$$X_i = G_{ii} + \frac{1}{2}\left(\sum_{j \neq i} G_{ij} + r\delta_i(G_{23} - G_{14})\right) \tag{A21}$$

where $\delta_i = 1$ for $i = 1, 4$ and $\delta_i = -1$ for $i = 2, 3$. We consider unidirectionnal

mutation from A_1 to A_2 and B_1 to B_2 at the same rate, u, so after mutation:

$$X_1^u = X_1(1-2u) \tag{A22a}$$

$$X_2^u = X_2(1-u) + X_1 u \tag{A22b}$$

$$X_3^u = X_3(1-u) + X_1 u \tag{A22c}$$

$$X_4^u = X_4 + (X_2 + X_3)u \tag{A22d}$$

After syngamy, we assume that genotype frequencies directly equilibrate to:

$$G_{ii}^r = (X_i^u)^2(1-F) + FX_i^u \tag{A23a}$$

$$G_{ij}^r = 2X_i^u X_j^u(1-F) \quad \text{for } i \neq j \tag{A23b}$$

And finally, after selection:

$$G'_{ij} = w_{ij} G_{ij}^r / \overline{W} \tag{A24}$$

where $\overline{W}$ is the mean fitness of the population. To simplify the system further, we can consider that intermediate haplotypes (A_1B_2 and A_2B_1) are maintained in approximate equilibrium at low frequency. This is true if $s >> u$ and $s >> r$. We also assume weak selection $s << 1$. Given the symmetry of the model $X_2 = X_3$ and are noted χ and we note $X_4 = x$, the frequency of the compensated haplotype, for which we want to calculate the probability and time to fixation. We can write:

$$\Delta\chi(\chi, x) = \Delta X_2 = \Delta X_3$$

$$\Delta x(\chi, x) = \Delta X_4$$

76 with the change of variables proposed above. We can use a separation of time

scale argument and consider that χ equilibrates much more rapidly than x.Thus we first solve $\Delta\chi(\chi, x) = 0$ for a given x and then plug the equilibrium χ value into $\Delta x(\chi, x)$. We thus obtain a an equation with a single variable that can be treated with classical diffusion theory. The full equation is not analytically tractable, however, noting that χ must be small, we can perform a Taylor expansion of $\Delta\chi(\chi, x)$ in χ at the first order and solve the resulting linear equation in χ. With the help of *Mathematica* we obtained:

$$\chi_{eq}(x) = \frac{(1-x)(u + x(1-F)(r - (h + F - hF)rs - hksu))}{s(h(1-2kx(1-x)) + F(1-h+2hkx(1-x)))} \tag{A25}$$

For $h = 0$ and $F = 0$ the first order term in χ vanishes so we need expansion at the second order, which gives:

$$\chi_{eq}(x) = \sqrt{\frac{(1-x)(rx+u)}{s}} \tag{A26}$$

Then we plug either (A25) or (A26) into $\Delta x(\chi, x)$. The full expression is rather cumbersome but it can be approximated as follows. As we assumed that all parameters are small: $u, s, r = O(\epsilon)$ with $\epsilon << 1$, we can only kept first order terms, which correspond to terms in s, r, u and u^2/s. With the help of *Mathematica* we obtained for $h > 0$ or $F > 0$:

$$\Delta x = \underbrace{2\frac{u^2}{(F+(1-F)h)s}(1-x)C_1}_{\text{Mutational input}} + \underbrace{x(1-x)\left(2uC_2 - (1-F)(khs(1-2x) + rC_3)\right)}_{\text{selection-like dynamics}} \tag{A27}$$

where C_1, C_2 and C_3 are expressions independent of s, r and u:

$$\begin{aligned}
C_1 &= \frac{1}{(1-2kx(1-x)))} \\
C_2 &= 1 - \frac{(1-F)hk(1-2x^2)}{F+(1-F)h(1-2kx(1-x))} \\
C_3 &= 1 - 2x\frac{(1-F)(F+(1-F)h(1+k(1-2x)))}{F+(1-F)h(1-2kx(1-x))}
\end{aligned}$$

The first term in equation (A27) corresponds to the input of the second mutation on the deleterious haplotypes, either A_1B_2 or A_2B_1 (hence the factor 2), which are both at mutation-selection balance equilibrium, $\frac{u}{(F+(1-F)h)s}$. The second term corresponds to selection-like dynamics of the form $Sx(1-x)$ where S has a complex form here. First, as the mean fitness of the population is of the order of $1-2u$ (see classical load theory, ex [8]), the fitness of the double mutant is simply of the order of $2u$ (but also depends on k and F). The second term corresponds to selection against double heterozygotes $(1-F)khs$ and breakdown of the double mutant by recombination $r(1-F)$. When $r=0$ and $k=0$, the double mutant A_2B_2 simply behaves as a beneficial mutations. On the contrary, the double mutant behaves as a deleterious mutations when recombination or selection overwhelm mutation:

$$(1-F)(khs + rC_3) > 2uC_2 \tag{A28}$$

So just a little recombination or selection against double heterozygotes greatly reduce the probability of fixation of the double mutant. From (A28) it is also clear that selfing increases the conditions of fixation of the double mutant.

A2.2 Probability and time to fixation

Equation (A27) can be injected in a classical one dimensional diffusion equation and numerically solved to obtain the time to fixation as in Kimura 1980 [4]. There is no analytical solution to the full equation but we can obtained a rather simple analytical approximation as follows. As in the main text, the time until ultimate fixation can be decomposed into the waiting time of the mutation destined to fixate and the time to fixation, conditional to fixation. The first term is usually much larger than the first one so we can only consider the waiting time and we can apply diffusion theory using (A7) and (A8) and modified version of (A11):

$$T_{fix} \approx \frac{1}{4Nu\chi_{eq}P_{fix}} \tag{A29}$$

because we only consider mutation arising on deleterious haplotypes, whose number is $2N\chi_{eq}$ in the population.

When $r = 0$ and $k = 0$, $C_1 = C_2 = 1$ and the selection-like term reduces to $2u$. So we have:

$$T_{0,0} \approx \frac{(F + h - hF)s}{2u^2} \frac{1 - e^{-8Nu/(1+F)}}{8Nu/(1 + F)} \tag{A30}$$

which reduces to:

$$T^*_{0,0} \approx \frac{(F + h - hF)s}{2u^2} \tag{A31}$$

when $4Nu < 1$ as given in the main text.

When $k = 0$ but $r > 0$, we still have $C_1 = C_2 = 1$ and $C_3 = 1 - 2x$ if $r > u$ we can neglect the mutation term so we obtain a simple selection like term:

$r(1-F)x(1-x)(1-2x)$. This leads to the following solution:

$$T_{r,0} \approx \frac{(F+h-hF)s}{2u^2} \frac{1}{N\left(1 - \frac{Erf\left((1-1/N)\sqrt{R}\right)}{Erf\left(\sqrt{R}\right)}\right)} \tag{A32}$$

with $R = N(1-\sigma)r$. Similarly, when $r = 0$ and $k > 0$ the selection-like term takes the same form, $hks(1-F)x(1-x)(1-2x)$ hence the same result as equation (A32) with $R = N(1-\sigma)hks$. This illustrates that recombination and selection against double heterozygotes play the same role. The general equation is more difficult to solve but as recombination and selection have the same form, so a heuristic argument is to use equation (A32) with $R = N(1-\sigma)(r+hks)$. Finally, to simplify the expression we can take the limit o (A32) when $N \to \infty$ (but $R \to cte$), which leads to:

$$T_{r,k} \approx \frac{(F+h-hF)s}{2u^2} \frac{\sqrt{\pi}e^R Erf(\sqrt{R})}{2\sqrt{R}}$$
$$\text{with} \quad R = N(1-\sigma)(r+hks) \tag{A33}$$

as given in the main text. Simulations show that this general approximation is rather accurate and allows a clear interpretation of the effect of recombination, selection against heterozygotes and selfing. It is important to note that these results are valid when effective recombination is low ($r(1-F)$). In the main text, simulations show that they quantitatively breakdown when recombination is too high. However these approximations are useful to characterize the effect of selfing on the fixation of compensatory mutations.

References

1. Pollak E. On the theory of partially inbreeding finite populations. I. Partial selfing. Genetics. 1987;117(2):353–60.

2. Caballero A, Hill WG. Effects of partial inbreeding on fixation rates and variation of mutant genes. Genetics. 1992;131(2):493–507.

3. Kimura M. On the probability of fixation of mutant genes in a population. Genetics. 1962;47:713–9.

4. Kimura M. Average time until fixation of a mutant allele in a finite population under continued mutation pressure: Studies by analytical, numerical, and pseudo-sampling methods. Proceedings of the National Academy of Sciences. 1980;77(1):522–526.

5. Glémin S, Ronfort J. Adaptation and maladaptation in selfing and outcrossing species: new mutations versus standing variation. Evolution: International Journal of Organic Evolution. 2013;67(1):225–240.

6. Kimura M. The role of compensatory neutral mutations in molecular evolution. J Genetics. 1985;64(1):7–19.

7. Stephan W. The rate of compensatory evolution. Genetics. 1996;144(1):419–426.

8. Charlesworth B, Charlesworth D. Elements of Evolutionary Genetics; 2010.
